# Agent‐Based Simulations of Lung Tumor Evolution Suggest That Ongoing Cell Competition Drives Realistic Clonal Expansions

**DOI:** 10.1002/advs.76764

**Published:** 2026-08-06

**Authors:** Helena Coggan, James R. M. Black, Carlos Martínez‐Ruiz, Kristiana Grigoriadis, Jasmin Fisher, Nicholas McGranahan

**Affiliations:** ^1^ Cancer Research UK Lung Cancer Centre of Excellence University College London Cancer Institute London UK; ^2^ Computational Cancer Biology Research Group University College London Cancer Institute London UK; ^3^ Department of Mathematics University College London London UK; ^4^ Cancer Genome Evolution Research Group University College London Cancer Institute London UK; ^5^ Cancer Evolution and Genome Instability Laboratory the Francis Crick Institute London UK

**Keywords:** agent‐based models, lung cancer, tumor evolution

## Abstract

Computational simulations of tumor evolution are increasingly used to infer the rules underlying cancer growth. To make reliable inferences, such models must be able to reflect the properties of real tumors. Recent work has shown that lung tumors undergo frequent and late subclonal expansions, which are associated with poor prognosis. This paper tests three candidate simulations of three‐dimensional tumor growth, which make different assumptions about the nature of competition between cells, for their ability to replicate these late expansions. The study identifies a computationally‐efficient model which can produce multi‐region sequencing data realistic to lung tumors. This model assumes two distinct stages of growth, with the second stage involving local competition for space and resources within and between small tissue areas in a fixed‐size tumor. When inferring the model‐specific fitness effect of driver mutations in a large cohort of lung cancers, the study finds that inference pipelines based on a two‐stage model imply much larger selection effects than those based on single‐stage models, driven by model‐specific assumptions about the practical consequences of selection strength. This work emphasizes the importance of model assumptions to the results of tumor‐specific, simulation‐based inferences.

## Graphical abstract

Computational simulations of tumor evolution are increasingly used to infer the rules underlying cancer growth, with the goal of one day recommending tailored treatments. Here we show that the properties of lung cancer sequencing data are best replicated by a model which assumes that cells compete both to proliferate and survive.

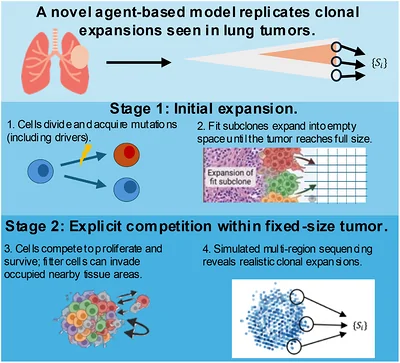

## 1 Introduction

In the last decade, spatial agent‐based models (SABMs) have become an increasingly popular tool for investigating the underlying rules of cancer evolution [1, 2]. Such models usually start with a single cell in the center of a two‐dimensional [3, 4, 5, 6, 7, 8, 9] or three‐dimensional grid [10, 11, 12, 13, 14], and simulate cell division, death and acquisition of mutations until the tumor is sufficiently large. At this point, the simulated tumor is sampled, and these samples are sequenced, to measure the number and frequency of the mutations present. These simulated data can then be compared to real tumor samples. In this way, SABMs can be used to infer the evolutionary rules driving the growth of specific tumors. This approach leverages the increasing availability of multi‐region sequencing (MRS) tumor data, which provide a clearer picture of the evolutionary trajectory of a tumor. MRS data shed light on the extent to which genotypes (point mutations, copy‐number alterations, structural variants, etc.) or phenotypes (RNA signatures, patterns of protein expression) are shared between regions, quantifying the genetic “relatedness” or phenotypic similarity of different parts of the tumor [8, 11]. If mutations are found in identical proportions of cancer cells in distant regions, the tumor can be said to be genetically homogeneous, with closely‐related samples colonized by a single cell population (or genetic “clone”). In contrast, if two samples share a relatively low proportion of their mutations, we can say they are less related, colonized by different “subclones” in a tumor experiencing ongoing diversification. By developing simulations to decipher the tumor‐type‐specific rules of cancer evolution, researchers can develop a deeper understanding of the rules and modes of tumor development, which may ultimately inform strategies to prevent and treat cancers.

To produce reliable inferences, however, the model must be able to reflect the biology of real tumors. Spatial models of evolution and competition are well‐established: Moran models have been used for over sixty years to study the dynamics of cancer initiation in well‐mixed populations [15, 16, 17], one‐dimensional population structures [18, 19, 20], lattices and graphs [21, 22, 23, 24, 25, 26, 27], and realistic Voronoi networks mimicking epithelial tissues [28, 29]. However, Moran models assume a fixed or nearly‐fixed population size and so are ill‐suited to model competition within an expanding tumor. To generate multi‐region sequencing data, we turn to agent‐based models, which treat cells as agents interacting according to fixed rules, extending our previous work [30].

Historically, SABMs of solid tumors have generally been modeled either on colorectal cancer (CRC) [6, 8, 10, 11, 31, 32, 33], due to the relative abundance of MRS datasets in CRCs, or have been tumor‐type‐agnostic [5, 6, 13, 14, 34, 35, 36, 37, 38, 39, 40, 41, 42]. In this study we are interested specifically in lung cancer, the leading source of cancer‐related death worldwide [43], with five‐year survival rates of around 20% [44]. Recently‐available MRS data from non‐small‐cell lung cancers (NSCLCs) have revealed patterns of intra‐tumoral heterogeneity suggestive of large, frequent regional expansions by “subclones” of related cells (illustrated in Figure 1). In particular, lung tumors are marked by frequent “clonal illusions” (CIs), mutations which are present in every cell (“clonal”) in at least one sample but are non‐ubiquitous (or “subclonal”) in other samples, suggesting a clone which has overtaken part but not all of a tumor. These regional subclonal expansions have been linked to poorer prognosis [45], and are suggestive of fierce competition between cancer cells. In this study we aimed to develop a three‐dimensional model which was capable of replicating realistic levels of clonal illusions, drawn from publicly available MRS data on a cohort of treatment‐naive lung tumors (the TRACERx [TRAcking Non‐small Cell Lung Cancer Evolution Through Therapy (Rx)] dataset [45]). We required our model to be computationally efficient (requiring no more than a few hours of runtime, less than 5GB of RAM and 100 GB of scratch space), so that it could be run efficiently in parallel on a high‐performance computing (HPC) cluster. We took as our starting point the 3D SABM developed by Hu and coauthors as a comparison to multiple tumor types [12], and sought to establish whether and how alterations had to be made in order to reproduce the clonal alterations seen in lung cancer, without compromising computational feasibility.

**FIGURE 1:**
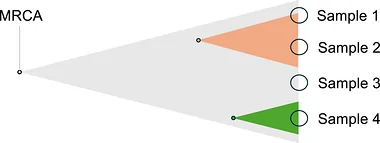
A schematic of the regional subclonal expansions characteristic of lung tumors in the TRACERx cohort. All cells in the tumor are descended from a single cell, the most recent common ancestor (MRCA, marked in gray). At the end of the tumor's growth, it is resected and subject to multi‐region sequencing. During tumor growth, certain lineages spread and fix in regions of the tumor without “sweeping” the entire lesion and becoming the MRCA. Mutations present in the cell marked in orange are detected in Sample 1, fixed (detected in all cells) in Sample 2 and absent from Samples 3 and 4. Mutations present in the cell marked in green are fixed and private in Sample 4.

All agent‐based models (ABMs) are developed with finite computational constraints in mind, and so all modelers are forced eventually to prioritize some aspects of tumor biology over others, which may be abstracted away or neglected entirely. This is especially true when encoding assumptions about cell competition. It is significantly cheaper to assume that a cell's ability to divide or die (its “fitness”) is an invariant across its lifetime, so that simulating division or death requires a fixed number of operations per cell, than it is to assume that fitness to depends explicitly on the number and fitness of other cells in its vicinity, which often requires explicit comparison of every cell to every other cell and forces constant recalculation of cell properties. Existing three‐dimensional ABMs have balanced this tradeoff in different ways. A common approach, which we replicate in Model 1, is to assume that a cell's fitness is time‐invariant (or absolute) but that cell populations compete for finite chunks of space or resources, which are awarded in a first‐come‐first‐served manner to the first cell population to reach a certain size [10, 11, 12]. Other approaches involve choosing the fundamental unit of the simulation to represent a clonal population or group of cells, rather than individual cells [8, 46]; mediating competition through local or global carrying capacity, rather than explicitly computing the actual number or fitness of competitor cells [34, 47]; or modeling a small number of cells explicitly and then “scaling up” to a full tumor through deterministic ordinary differential equations [48]. By choosing not to represent competition between individual cells explicitly or across the entire tumor, these models can afford detailed representations of, for example, tumor‐normal or tumor‐immune interactions, explicit cell cycles, or increased spatial granularity.

In this work we focus on the tradeoff between the representation of complex mechanisms of competition and the overall size of the simulated tumor. Because we are interested primarily in replicating clonal illusions, we design simulation frameworks in which mutation, migration, division and death are simulated at the level of individual cells. To keep the model computationally feasible, we make a number of simplifying assumptions about other aspects of tumor biology. This explicit representation of mutational prevalence allows us to generate realistic simulated whole‐exome sequencing (WES) data, which enables the comparison of the outputs of each model to the properties of lung tumors in the TRACERx cohort. Our aim is to evaluate the complexity of cell competition required to simulate the clonal expansions seen in lung tumors.

Within these conceptual and computational constraints, we build three different modeling frameworks. In Model 1, based on SCIMET (Spatial Computational Inference of MEtastatic Timing) [11, 12], we assume that driver mutations increase a cell's proliferation rate and decrease its death rate, both of which are independent of a cell's local environment. In this model, cells compete implicitly in a first‐come‐first‐served manner for space on a three‐dimensional grid. Model 2 introduces “active competition” [49], such that a cell is less likely to die when it is fitter than other cells in its immediate environment. Model 2 thus combines both continuous tumor growth and local competition, but comes at the cost of a smaller final tumor size and fixed overall tumor growth rate. Model 3 takes this a step further and divides tumor growth into two stages: an initial “exponential” stage similar to Model 1, and a “homeostatic” stage in which cells compete for both proliferation and survival within a fixed‐size tumor, both within and between local tissue areas. This model represents the most complex and stringent set of rules around competition, but is the most computationally arduous and imposes the smallest final tumor size.

We vary the selection strength and death rate within each model. Amongst the models and parameter ranges tested, we find that a high‐selection regime within Model 3, in which cells compete for survival both within and between local tissue areas, is best able to replicate the late, region‐specific clonal expansions characteristic of NSCLC tumors in the TRACERx cohort (Section 3). We also examine how accurately selection can be inferred from simulated data, leveraging methods from machine learning and Approximate Bayesian Computation (ABC) (Section 3.3), and demonstrate that choice of underlying model has a profound impact on the selection strength inferred from TRACERx tumors.

This study presents a novel two‐stage, three‐dimensional agent‐based model, designed to generate realistic whole‐exome sequencing data from simulated lung tumors. Unlike previous models, we represent explicit, multilevel cell–cell competition for both proliferation and survival, and do so in a model lightweight and efficient enough to be run in parallel on an HPC many thousands of times in a few hours. The models rely on a series of design choices motivated by replicating bulk multi‐region summary statistics, such that mutations are calculated from ancestry records only at the end of the simulation and then only to cells which form part of the final simulated samples. This choice means that records of the mutations held by individual cells in a 3D tumor do not need to be stored in RAM, massively increasing simulation efficiency. We benchmark our model's performance against TRACERx data using a custom‐built, machine‐learning (ML)‐driven Bayesian inference pipeline. Our work highlights the importance of model assumptions to the ability of lung‐cancer‐specific ABMs to replicate the properties of real tumors.

Our code is written in Python, and is publicly available in a Github repository (hcoggan/Agent‐based‐simulations‐of‐lung‐tumor‐evolution, release tag march‐2026).

## 2 Model Design

We present three candidate models of tumor evolution. All three assume that changes in cell fitness arise as a result of somatic driver mutations of uniform selection strength s, though the precise effect of this selection strength differs between models. At the end of the simulation, eight small samples are taken from the surface of the tumor and subjected to realistic whole‐exome sequencing (see Figure 2).

**FIGURE 2:**
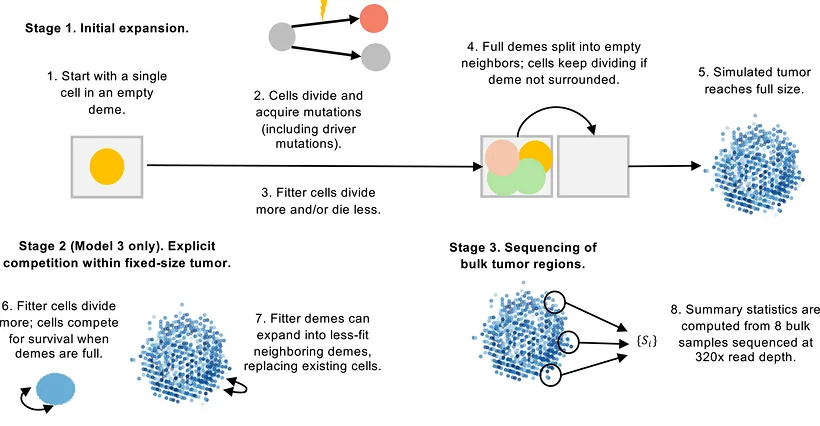
A schematic of the general modeling framework. Specific rules governing migration and death vary between models. ‘Lightning bolts’ indicate the acquisition of a genetic alteration.

Models share a common set of assumptions about spatial structure, mutation rate and three‐dimensional growth dynamics. Each model makes different assumptions about the effect of selection on a cell's proliferation or survival. In Model 1 (adapted from SCIMET [12]), cells are increasingly likely to divide as they acquire driver mutations. Cells compete *implicitly* for limited space and resources (in the sense that a cell's fitness is solely a function of its own driver mutations), but not *explicitly* (in the sense that a cell's ability to survive or proliferate is an explicit function of the fitness of other cells in its immediate environment, not merely its own fitness). The two subsequent models introduce increasingly severe competition for space and resources. In Model 2, cells compete explicitly for survival within their local environment as the tumor continues to expand. In Model 3, the tumor expands to a fixed size and then continues to evolve during a second ‘homeostatic’ stage of growth, in which cells compete explicitly for survival both within *and* between occupied tissue areas.

A brief overview of the differences between models is provided in Table 1. A full list of parameters used in each model is given in Table 2. The workings of each model are discussed below. Further details can be found in Supporting Information.

**TABLE 1:** The mechanistic differences between models presented in this paper.

	Model 1	Model 2	Model 3
Local competition	Implicit	Explicit (for survival)	Explicit (for proliferation and survival)
Competition between tissue areas (for new tissue)	Implicit	Explicit	Implicit
Competition between tissue areas (for occupied tissue)	No	No	Yes
Stages of growth	1	1	2
Death rate	Variable	Variable	Fixed
Final tumor size (in millions of cells)	500	100	5

**TABLE 2:** A list of parameters used in each model.

Parameter	Model 1	Model 2	Model 3	Source
Size of tumor	500 million cells	100 million cells	5 million cells	Computational constraints
Age of tumor	40 days–5 years	40 days–5 years	1 year	Consultation with colleagues
Baseline probability that a cell will die per timestep $\left(\lambda_{0}\right)$	0.0, 0.1, 0.2, 0.3, 0.35, 0.38	0.0, 0.1, 0.2, 0.3, 0.35, 0.38	None until deme full	N/A (variable parameter)
Selection strength s	Varied between 0–0.1			[10, 11]
Mutation rate μ (average exome mutations per division)	0.4			[57]
Size of local environment, $N_{0}$ (“deme”)	10,000 cells			[11, 57]
Size of Moore neighborhood	26 demes			[2]
Size of timestep δt	1 day			Consultation with colleagues
Probability that a mutation is a driver, θ	$10^{-5}$			[52]
Probability per timestep that the founding cell will divide, $p_{0}$	0.631			[54, 55] (see text)
Maximum number of samples, M, from any tumor	8			[45, 57]
Minimum CCF required for detection of a mutation in a sample	0.01			[45]
Minimum number of reads required for detection of a mutation in a specific sample	4			[45]
Minimum number of reads in any sample required for detection of a mutation	10			[45]
Sequencing depth	320× (400× at 80% purity)			Consultation with colleagues
Sequencing error rate, ε	0.0001			[57]
Cells per bulk tumor sample	50,000			[57]

### 2.1 Mutation

All simulated tumors begin as a single transformed cell. We are interested in modeling only the acquisition and spread of mutations during tumor evolution. As a result, we ignore mutations accumulated prior to tumorigenesis and concern ourselves only with the mutations acquired during tumor growth.

When a cell divides, each daughter cell acquires a number of exome mutations, $n_{\mu }\geq 0$. Cells may acquire multiple mutations on division. $n_{\mu }$ is Poisson‐distributed with mean μ=0.4 (toward the lower end of the range provided in ref. [50]). We treat all mutations as identical and do not consider distinctions between “point mutations” (SNVs), copy‐number alterations, structural rearrangements and whole‐genome doubling (WGD) events. We use the infinite‐sites assumption [51], so that no mutation can occur more than once or be lost after it has been acquired, following well‐established convention [6, 8, 10, 35].

Somatic mutations are divided into two categories. A mutation is a “driver” with probability $\theta =10^{-5}$ (a common literature estimate originating with the work of Bozic et al. [52]), and confers a fitness boost of strength s. (We assume that cells can acquire at most one new driver mutation on each division, such that if a cell which has acquired $n_{\mu }$ new mutations, one of them is a driver with probability $n_{\mu }\theta$.) All other mutations are neutral, on the assumption that positive selection strongly outweighs the deleterious effects of passenger mutations [53].

We note here that the computational feasibility of our modeling framework lies, in part, in the way that ancestry is encoded. Any unique set of mutations defines a “genotype”; when mutations occur during a cell division, a new genotype is born. We represent genotypes as unique consecutive integers, and store the identities of mother‐daughter genotypes in a dictionary in global memory, along with a record of the number of mutations separating them. This allows us to minimize RAM requirements, as a cell can be represented with only two integers: its genotype and the number of driver mutations it carries. All models make use of a custom‐built algorithm which reconstructs the full ancestral tree only at the end of the simulation and *only for cells that are sequenced*.

The decision to leave the mutational identity of most cells implicit, and then only compute the full set of mutations for each cell where necessary, means that the space required to represent cells does not increase as they acquire passenger mutations, which would render the simulation immediately infeasible at scale. The downside to this approach is that the full clonal identity of all cells in the tumor remains unknown, as reconstructing the ancestral tree for the entire tumor would increase the computational burden by 1–2 orders of magnitude. It is also infeasible to continually track clonal expansions over time, as this would require ancestral reconstruction not merely of every cell but *at every timestep*. This tradeoff allows better representation of single‐cell dynamics and whole‐exome sequencing at scale.

### 2.2 Spatial Structure and Cell Migration

All models divide space into voxels referred to as “demes.” Demes conceptually represent local tissue areas, and are assumed to have a maximum capacity of $N_{0}=10000$ cells [10, 11, 12] for the sake of computational efficiency. Inter‐deme competition (represented in Models 2 and 3) scales with the number of demes in the model and so is made cheaper by coarser spatial graining (i.e., larger deme sizes). This limit ensures that even in the smallest model considered here (e.g., in Model 3, which has a final tumor size of 5 million cells), an individual deme represents only 0.2% of the tumor's population.

Demes are arranged on a three‐dimensional grid, governed by a Moore neighborhood with 26 neighbors. Within demes, cells are assumed to be spatially well‐mixed. Competition for space and resources takes place within demes. If a deme's population N is at capacity ($N\geq N_{0}$) this generally imposes limits on the ability of cells to divide, and may force cell death or migration. In models in which cells compete explicitly for survival or proliferation (Models 2 and 3), in the sense that a cell's likelihood of division or death is explicitly dependent on the fitness of other cells in its environment, competition is limited to cells in the same deme. This encodes the idea that cell fate depends on local conditions (density, available nutrients, etc.), and that information travels much more slowly between distant tissue areas. The limited capacity of demes acts as a “carrying capacity,” abstracting away the granular processes of nutrient uptake, angiogenesis, mechanical pressure, competition with normal cells, and immune interactions.

Over the course of the simulation, the tumor expands through three dimensions as cells colonize new demes. During a colonization event, the population of the focal deme is split in half at random, and one half moves into a neighboring deme. Cells in demes which are completely surrounded (i.e., which have no empty neighboring demes) are assumed to die immediately, as they cannot receive the necessary nutrients for survival. All information about the genetic identity of these dead cells is immediately deleted, ensuring the simulation is lightweight and memory‐efficient.

All models begin with a single founding cell in the central deme, and the simulation proceeds in timesteps of 1 day. All demes other than the central deme are initially empty, and normal cells are not explicitly modeled. Model‐specific colonization rules are summarized briefly below; full details are provided in Supporting Information.

#### 2.2.1 Model 1

In Model 1, colonization happens whenever an individual deme becomes full (i.e., the current population of the deme, N, is such that $N>N_{0}$ after division and death). Full demes split into a randomly‐chosen neighbor, if one exists.

#### 2.2.2 Model 2

In Model 2, colonization happens in “rounds,” whenever all active demes (i.e., those with empty neighboring demes) are full. During a colonization “round,” full demes compete for the opportunity to seed into empty space, with a probability of success proportional to their relative fitness (defined below).

#### 2.2.3 Model 3

In Model 3, tumor growth proceeds in two stages. During the first, “exponential” stage (before the tumor has a total population of 5 million), colonization proceeds exactly as in Model 1.

In the second “homeostatic” stage, the tumor maintains a fixed size, and competition begins between active demes. At each timestep, an active deme may seed into a less‐fit neighboring deme, a process which is assumed to kill all cells in the invaded deme. The probability of invasion is proportional to the ratio of the average fitness of the cells in each deme. The definition of “fitness” is discussed below.

### 2.3 Cell Proliferation

As cells evolve, they acquire driver mutations, which confer boosts to cell fitness. These boosts have size s, a simulation parameter referred to as the selection strength. These fitness boosts always affect proliferation‐fitness, but only affect cell *survival* in Models 2 and 3 (see section below).

In all models, proliferation‐fitness evolves as follows. If we assume that $p_{0}$ is the probability that the founding cell will divide in a given 1‐day timestep, we assume that cell with k drivers will divide with a per‐timestep probability

$$p_{p}\left(s,k,p_{0}\right)=p_{0}+\left(1-p_{0}\right)\left(1-{\left(1-s\right)}^{k}\right)$$

This proliferation‐fitness $p_{p}=p_{0}$ where a cell has no drivers, or where those boosts are ineffectual (i.e., for s=0 or k=0). As the number of boosts increases, however, the probability of division approaches 1 asymptotically. This ensures that each additional boost has a (diminishing) effect on proliferative fitness, whilst preventing nonsensical probabilities of division (i.e., p>1). This probabilistic division scheme allows cells to have variable mitotic ages– an improvement on existing three‐dimensional simulation frameworks designed for tumor‐specific inference [11, 12], which assume that cells either divide or die at every timestep.

Our initial cell has a cell cycle time of 24 h. This is on the lower end of literature estimates for NSCLC cell lines [54, 55], on the assumption that cells are less well‐adapted and thus slower to divide in the early stages of tumor evolution. This corresponds to an initial proliferative fitness (probability of division per day) of $p_{0}=0.631$ for the founding cell.

All driver mutations in a tumor are assumed to have the same effect on cell fitness. This ensures that fitness is consistently recalculable from the record of the integer number of drivers possessed by each cell and the global fitness s, which keeps memory requirements reasonable.

The fitness effects of driver point mutations are generally considered to be in the range 1%–10% [11, 12, 56]. In this study we explore selection values in the range 0≤s≤0.1. However, we emphasize that s=0.1 does not translate straightforwardly to a 10% increase in growth rate (as it might in an exponential model). Nor does it correspond to a 10% increase (decrease) in the likelihood of stochastic division (death), as it might in a discrete‐time birth‐death model. This is in part because of the saturating effect of drivers on proliferation fitness discussed above, and in part because the effect of selection on survival‐fitness (discussed in Section 2.4), which varies between models. In Models 2 and 3, which encode explicit competition for survival between cells, the effect of each additional driver mutation on a cell's likelihood of death depends not just on its genotype but on the local fitness landscape, and so the effect of selection on growth rate becomes extremely difficult to calculate. Intuitively, we can think of the effect of selection as approximately multiplicative, so that every new driver makes a cell “fitter” by a factor of s; but in general the quantitative effect of selection is not precisely comparable between models, as it is a function of the underlying assumptions.

### 2.4 Cell Death and Competition for Survival

In all models, driver mutations affect the likelihood that a cell will die in any given timestep (survival‐fitness), as well as its propensity to divide (proliferation‐fitness). Unlike proliferation‐fitness, the operation of survival‐fitness is model‐specific.

In Model 1, a cell's fitness is an inherent function of its genotype, and cells compete implicitly for space and resources. In Models 2 and 3, cells compete explicitly for survival: a cell's death rate is no longer inherent, and depends on the fitness of other cells in its vicinity.

#### 2.4.1 Model 1

Cells without driver mutations have a baseline probability $\lambda_{0}$ of dying per timestep. This death rate is a variable simulation parameter. Cells with more driver mutations tend to die less, so that a cell with k driver mutations (with selection strength s) dies with a per‐timestep probability λ given by:

$$\lambda \left(s,k\right)=\lambda_{0}{\left(1-s\right)}^{k}$$

We explore values of $\lambda_{0}$ in the range [0.0−0.38], with the upper limit chosen to result in an overall growth rate (of the founding lineage) of 1% per timestep, which experience suggests is the minimum necessary to prevent stochastic “stalling” of tumor growth (see Supporting Information).

#### 2.4.2 Model 2

In Model 2, a cell's death rate is controlled both by the global death rate and by the fitness of cells in its immediate vicinity (i.e., its deme). A cell with k driver mutations has “survival‐fitness” $f_{s}={\left(1+s\right)}^{k}$.

For a baseline death rate of $\lambda_{0}$, a cell's probability of dying in a given timestep is given by

$$\lambda =\max(1-\left(1-\lambda_{0}\right)\frac{f_{s}}{{\bar{f}}_{s}},0)$$

where ${\bar{f}}_{s}$ represents the average fitness of all cells in the deme. The average cell therefore dies with probability $\lambda_{0}$ per timestep, and cells which are fitter (less fit) than their local competitors are less (more) likely to die.

#### 2.4.3 Model 3

During the first “exponential” stage of growth, cells do not die. In the second “homeostatic” stage, death rates in a deme are dependent on the space available, rather than controlled by an overall death rate. After cells in a deme have proliferated, death occurs probabilistically only if the total population of the deme N is greater than its capacity $N_{0}$.

As in Model 2, a cell with k drivers of strength s has survival‐fitness $f_{s}={\left(1+s\right)}^{k}$. A cell with survival‐fitness $f_{s}$ dies with probability $\lambda =\max(0,1-\frac{N_{0}f_{s}}{F})$, where F is the total survival‐fitness of all cells in the deme. If the deme contains N cells of equal fitness, then their probability of survival will each be $N_{0}/N$, and we will expect roughly $N_{0}$ cells to survive at the end of the step. Thus, cells in a deme compete on fitness for sufficient space and resources, with fitter cells likelier to survive.

### 2.5 Realistic Sampling and Sequencing

The increasing complexity of the competition dynamics simulated in each model results in decreasing maximum computationally feasible tumor size (500 million for Model 1, 100 million for Model 2, and 5 million for Model 3).

At the end of the simulation, we take 8 spatially separated samples. This is the maximum number obtained from any tumor in the TRACERx421 dataset [45], which comprises 382 tumors with a median of 3 sampled regions (inter‐quartile range 2.0–4.0; see Figure S4). To ensure that variations in mutational frequency reflect *intra‐tumor* heterogeneity, we exclude tumors with fewer than 2 samples, as well as samples which initially appeared to have been taken from the same tumor but were later discovered to comprise genomically distinct tumors in the same patient.

The locations of samples are determined by randomly generating 20 000 possible combinations of eight central demes, and then choosing the set of samples with the maximum total squared distance between centers. This models the process by which samples are chosen in reality: human pathologists aim to maximize the spatial separation between samples, but do so approximately, by eye. Each sample comprises 1% of the tumor's surface cells (i.e., 1% of active demes). To replicate the sample sizes from the TRACERx cohort [57], and to ensure that models with larger final tumor sizes do not yield larger and more diverse samples, we down‐sample to roughly 50 000 cells (so around 400 000 cells in total from all samples). We then compute the ancestral tree for sampled cells, assign mutations to each cell, and compute the true prevalence (or cancer‐cell fraction, CCF) associated with each mutation in each sample.

We then perform simulated whole‐exome sequencing of each sample. We assume a tumor purity of 80% (such that 20% of reads are wasted on non‐malignant cells), and a total read depth of 400x, translating into an ‘effective’ read depth of R= 320x on tumor cells. We further assume an error rate of 0.01%; and a minimum detectable sample prevalence of 1% for all mutations.

Once a set of samples has been chosen, we filter mutations in accordance with TRACERx stringency protocols: mutations which have fewer than 10 reads in all individual samples are excluded (i.e., to pass this step, mutations must have a detected CCF of 6.25% in at least 1 sample).

Finally, before calculating summary statistics, we exclude all truncal mutations, defined as mutations with a detected CCF of 1.0 in all samples. In real tumors, it is impossible to tell whether truncal mutations occurred during tumor evolution or were present in the pre‐transformed cell. Therefore when comparing simulated tumors between models, and when comparing simulated to real tumors, we consider only subclonal mutations in order to compare “like with like.”

### 2.6 Calculating Summary Statistics

For a (simulated or real) tumor with M samples (where 2≤M≤8), we calculate the following 2M+1 summary statistics. Our key summary statistics are noted in bold.

1. Tumor mutational burden (**TMB**): the number of mutations detected in total, across the tumor.
2. The number of mutations detected in exactly 1,…,M samples (abbreviated to A1, A2, ...). (Mutations detected in only 1 sample are often referred to as “private.”)
3. Private tumor mutational burden (**TMB‐priv**): the number of mutations only detected in 1 sample.
4. Private fixed mutations (**F‐priv**): the number of mutations only detected in 1 sample, and “fixed” (i.e., with a detected CCF of 1.0) in that sample.
5. The number of mutations which are fixed in exactly 1,…,M−1 samples (abbreviated to F1, F2, ...). (Mutations fixed in M samples are clonal and thus excluded.)

We define a **clonal illusion (CI)** as a mutation which is fixed in *at least one region*, but is subclonal across all samples considered together. As all truncal mutations are excluded from our main summary statistics, this latter condition is automatically satisfied. The number of clonal illusions in a tumor with M samples is therefore

$$CI=\sum_{n=1}^{M-1}F\left(n\right)$$

where F(n) is the number of mutations fixed in exactly n samples.

## 3 Results

### 3.1 Comparing Simulated to Real Tumors With Multi‐Region Sequencing Data

We first assess the ability of each model to replicate the properties of lung cancer. We use three metrics to compare simulated tumors to cancers. First, for each summary statistic $S_{i}$, we can measure the distribution of statistics $S_{i}\left(s,M\right)$ produced by tumors with selection strength s, and M observed samples. (For Models 1 and 2, we can also vary cell death rate; see Supporting Information). We are interested initially in whether the average of $S_{i}\left(s,M\right)$ is “TRACERx‐plausible,” i.e., whether it falls within the 90% confidence interval for the corresponding summary statistic in comparable real tumors (i.e., those from which M samples were taken). We are also interested in whether we can reproduce the *median* value of that summary statistic in the TRACERx cohort, i.e., whether models can capture the properties of “average” lung tumors and not simply the extremes.

We focus on two key summary statistics: the simulated tumor mutational burden (TMB), the number of mutations observed across the tumor (Figure 3); and the number of clonal illusions (CIs), mutations which are fixed (CCF = 1.0) in at least one region but not all (Figure 4).

**FIGURE 3:**
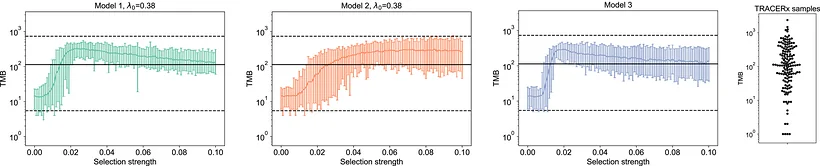
The variation of simulated tumor mutational burden (TMB) in each model with selection strength s. Models 1 and 2 have an assumed baseline death rate of $\lambda_{0}=0.38$. We assume M=2 samples. Averages are taken from 100 repeats at each s value, with maxima and minima indicated by error bars. Dotted black lines indicate the outer limits (5th and 95th percentiles) of corresponding summary statistics from all TRACERx tumors which were sampled at M regions. Solid black lines indicate the medians of these real TRACERx tumor cohorts. The rightmost panel is a stripplot of the distribution of the TMB in the real data.

**FIGURE 4:**
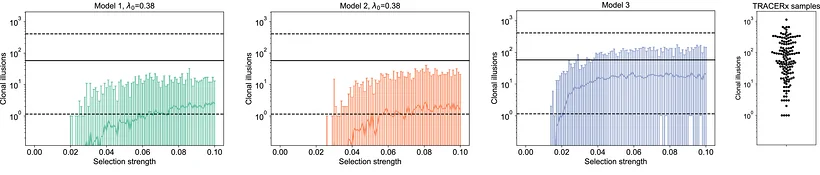
The variation of simulated clonal illusions (CIs) in each model with selection strength s. Models 1 and 2 have an assumed baseline death rate of $\lambda_{0}=0.38$. We assume M=2 samples. Averages are taken from 100 repeats at each s value, with maxima and minima indicated by error bars. Dotted black lines indicate the outer limits (5th and 95th percentiles) of corresponding summary statistics from all TRACERx tumors which were sampled at M regions. Solid black lines indicate the medians of these real TRACERx tumor cohorts. The rightmost panel is a stripplot of the distribution of CIs in the real data.

We can also consider $N_{truncal}$, the number of mutations observed at a CCF of 1.0 in all samples (and thus excluded from the calculation of all other summary statistics, $S_{i}$). As discussed, the presence of an unknown number of somatic mutations in the founding cells of real tumors makes it impossible to usefully compare $N_{truncal}$ between real and simulated tumors. However, differences in $N_{truncal}$ *between* simulated tumors can still be informative about the underlying model dynamics. In particular, in a realistic model of lung cancer, increasing selection strength should allow later‐arising lineages to dominate the tumor, and so extend the latest possible point at which a driver mutation can arise and still become the ancestor of all sampled cells. Higher selection should drive more and later “clonal sweeps,” or colonization of a tumor region by a late‐arising clone. This should mean that the most recent common ancestor (MRCA) has more mutations relative to the founding cell; thus $N_{truncal}$ should increase with s. We examine this in Figure 5.

**FIGURE 5:**
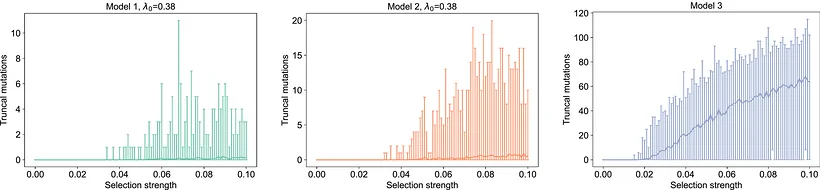
The variation of the number of truncal mutations, i.e. those detected at a cancer cell fraction (CCF) of 1.0 in every region (and are thus excluded from the calculation of all other summary statistics) in each model with selection strength s. Models 1 and 2 have an assumed baseline death rate of $\lambda_{0}=0.38$. We assume M=2 samples.

#### 3.1.1 Intra‐Tumoral Heterogeneity is Greatest at Intermediate Levels of Selection

We first observe that for models with a variable death rate (Models 1 and 2), realistic TMBs are generally only achieved for the highest possible baseline death rate ($\lambda_{0}=0.38$, corresponding to growth rates of 1% per day). This would imply that tumors have a final age of around 5 years; previous estimates have suggested that both lung adenocarcinomas (LUADs) and lung squamous cell carcinomas (LUSCs) are diagnosed roughly 2 years after the development of the most recent common ancestor (MRCA) [58], an event which occurs within the timeframe of our model (which begins post‐transformation).

Even where selection is strong, TMBs remain resolutely low for baseline death rates below the maximum at $\lambda_{0}=0.38$ (see Supplementary Figures 8 [Model 1] and 9 [Model 2]). This dependence on death rate is fairly intuitive: an increased $\lambda_{0}$ results in higher rates of cell death, which increases the time taken for tumors to reach full size and thus the mitotic age of the sampled cells. Cells acquire mutations when they divide, so this higher death rate leads to a higher TMB. A higher baseline death rate also increases the selective survival benefit (i.e., decrease in death rate) of driver mutations, and so will accelerate the pace of evolution.

**FIGURE 6:**
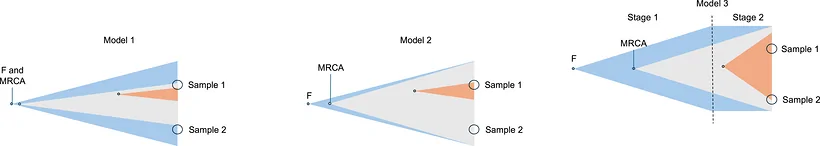
A fish plot providing a conceptual visualization of the growth patterns resulting from each model. The gray and red clones are successively fitter than the founding lineage F (blue clone). In Model 1, the gray clone increases in frequency but never fully eliminates the blue clone, and is found subclonally in both samples. The red clone appears later during tumor growth and is found subclonally in one sample. In Model 2, the gray clone arises early enough to eliminate the blue clone, becomes the most recent common ancestor (MRCA) of the tumor, and is observed clonally in both regions. The red clone, though fitter, arises too late to seed both samples and is observed (clonally or subclonally) in Sample 1. In Model 3, the red clone arises late in tumor growth (after the tumor reaches a fixed size, a point represented by the dotted line between stages) and is able to take over Sample 1 (so is detected clonally there) and spread to Sample 2 (so is detected subclonally).

**FIGURE 7:**
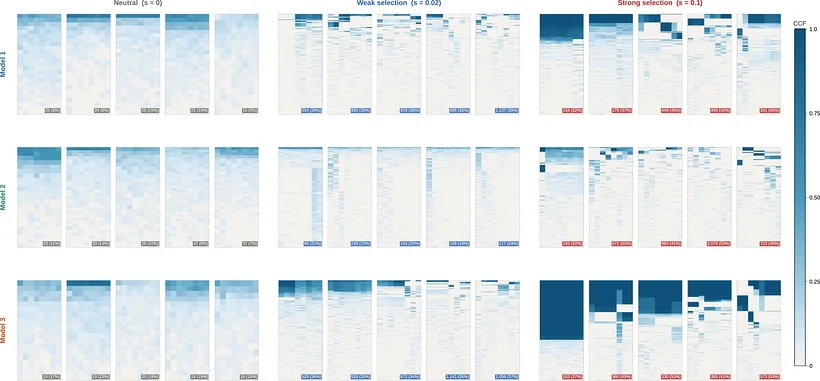
Multi‐region clonal cancer‐cell‐fraction (CCF) heatmaps across simulated tumor models. Rows of panels correspond to the three tumor growth models (Models 1, 2, and 3); columns of panels correspond to representative selection strength regimes (neutral, s=0.0; weak selection s=0.02; strong selection, s=0.1). Within each model × selection‐strength group, five representative simulations are shown. Each heatmap shows one simulated tumor: rows are individual somatic mutations, sorted from highest to lowest mean CCF (top to bottom); columns are the eight simulated multi‐region tumor samples, reordered within each simulation by hierarchical clustering on CCF similarity. Mutations with CCF < 5% in every sample were removed prior to plotting; the white‐to‐gray end of the color scale therefore represents non‐detection in a given sample among the remaining mutations, not the full unfiltered mutation set. Cell color encodes CCF from 0 (pale gray, not detected) to 1 (dark teal, fully clonal in that sample). The badge in the lower‐right corner of each heatmap reports the number of mutations retained after filtering and this number as a percentage of the total number of mutations simulated for that tumor.

**FIGURE 8:**
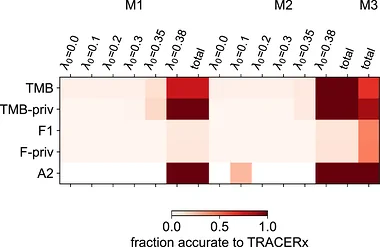
A comparison of the suitability of all 3 models to the TRACERx data, assuming M=2 samples. The element in the ith row and jth column indicates the probability that a simulation from model j will produce a summary statistic i within the 90% confidence interval of TRACERx tumors with M samples. Models 1 and 2 are broken out by death rate; the ‘total’ column displays the average across all death rates. TMB: tumor mutational burden; TMB‐priv: private tumor mutational burden; F1: clonal illusions; F‐priv: number of mutations detected only in 1 sample and fixed in that sample; A2: number of mutations detected in both samples.

Once we fix $\lambda_{0}=0.38$ for Models 1 and 2, all models can produce an equally realistic range of TMBs (see Figure 3), independently of sample number (see Figures S5 and S6). This is to say that different assumptions about competition do not strongly affect our ability to produce *diverse* tumors (using TMB as a measure of diversity).

The relationship between TMB and selection strength can also inform our understanding of the emergent growth dynamics in each model. In Models 1 and 3, cells either do not compete explicitly for survival (1) or compete only to recolonize existing tissue areas (3), but do not compete explicitly for new space. In both models, we observe that selection strength first increases and then decreases the mean TMB. First, we observe a peak TMB at weak selection (s≈0.02) (see Figure 3, left panel). For lower selection levels (s<0.01), competition is weak, and so very few mutations reach detectable prevalence; those which do are likely to have occurred early, and thus appear in multiple samples, such that the resulting tumor is very homogeneous. As competition increases, the sampled population begins to diversify. Selection is now strong enough to allow lineages with late‐occurring driver mutations to increase in frequency: sampled populations are composed of a large number of highly proliferative lineages, frequent enough to be detectable but not sufficiently advantaged to take over an entire region.

As selection strength increases further (s>0.02), the TMB begins to decrease again. Selective advantages are now strong enough that fitter lineages proliferate very quickly. These lineages invade empty space aggressively enough to block less‐fit lineages from reaching the surface. If this process occurs sufficiently early, these cells become the ancestors of multiple samples, increasing the genetic relatedness of sampled regions. This makes it less likely that any sampled mutation will be private, and decreases the overall number of genetic mutations (TMB) contained in the sampled regions. Similar patterns are observed in the number of mutations observed only in 1 tumor region (TMB‐priv; see Figure S7).

In Model 2, however, selection strength has a saturating effect on the TMB. tumor diversity is very low at s=0.0, increases slowly as selection increases, and then plateaus after s=0.05. This suggests that explicit competition for new space introduces a “bottleneck” on selection: beyond s=0.05, selection strength does not increase the diversity of the tumor. This in turn implies that there is a point in tumor growth at which it is simply too late for any new lineage, however fit, to reach detectable prevalence. This limits the ability of Model 2, which assumes that cells compete for expansion into new tissue but not into already‐colonized tissue, to replicate the competitive dynamics of lung cancer.

#### 3.1.2 Competition for Existing Space Allows Simulation of Late Subclonal Expansions

We next investigate the number of CIs observed in simulated tumors. If no CIs (or almost none) are observed, we can say that TRACERx‐like subclonal expansions do not occur in the model.

We observe that Models 1 and 2 are unable to replicate realistic numbers of clonal illusions at any selection level (see Figure 4). CIs are almost non‐existent for s<0.05 (i.e. with a mean of fewer than one per simulated tumor) and essentially constant for s≥0.05. This pattern is independent of the number of sampled regions M (Figures S12 and S13).

These results suggest that implicit competition (as in Model 1) or competition for new space alone (as in Model 2) are insufficient to allow late‐arising, fit lineages to take over individual regions before the tumor is sampled. On average, these models produce fewer than 10 clonal illusions per tumor, regardless of sample number. This is true even at high selection levels, where the TMB and TMB‐priv *are* in line with those of observed tumors. This suggests that we could not obtain competitive evolutionary dynamics under this model, even if it were computationally feasible to simulate larger tumors: the speed at which mutations spread is simply too slow relative to the rate at which the tumor diversifies. This leads us to a two‐stage model of growth in which cells compete for *existing* space after an initial expansion (Model 3).

Model 3 produces realistic levels of clonal illusions at high selection levels (s>0.02) (see Figure 4, right panel). As with the TMB (Figure 3), the number of clonal illusions increases with ongoing selection, and then saturates at a certain selection level (roughly s=0.04). We infer from this that mutations arising later in tumor evolution can spread beyond their sample of origin, so functional competition is ongoing and each increment of selection provides a continuing advantage. Continuing evolution allows lineages to spread through regions in a fixed‐size tumor.

Simulations are more likely to produce sufficient numbers of clonal illusions at low sample number (see Figures S12 and S13, bottom panels). In both real and simulated tumors, clonal illusions increase with sample number, because mutations which initially appeared truncal (because they had fixed in one or two samples) are revealed to be subclonal with additional sampling (because they are absent or unfixed in a subsequent sample). Our simulations appear to slightly underestimate the rate of this increase, perhaps because of computational limitations on simulated tumor size: larger tumors may be more spatially diverse than our simulations are currently able to capture. As a result, simulations produce ranges of clonal illusions at the lower end of the observed data (on average fewer than 100, regardless of sample number, when hundreds would be permissible). The *average* number of simulated clonal illusions, whilst within the 90% confidence interval for the TRACERx dataset, is always lower than the median for real tumors; amongst the model classes tested here, realistic values are produced only by ‘extreme’ simulations. Model 3 nonetheless represents a high‐water mark for our ability to replicate the properties of real lung cancers.

To test the dependence of this finding on the size and age of the modeled tumor, which are imposed at scale by computational constraints, we simulated a small number of tumors with high (s=0.1) selection and varying age (100 repeats each; see Tables S1 and S2). We found that Model 3 tumors are still able to produce more than 10 CIs on average when their age is halved or doubled (a mean of 11 CIs at 6 months; 16 CIs at 12 months, 21 at 18 months, and 26 at 24 months). Similarly, when the final size of the tumor was varied, the order of magnitude of clonal sweeps in Model 3 tumors was unchanged (a mean of 14 CIs at 2m cells, 19 at 5m, 28 at 10m, and 44 at 20m). We also find that varying the tumor size and age produces TMBs in the TRACERx‐appropriate range ($10^{1}-10^{3}$); the mean TMB is particularly robust to this variation, increasing only from 117 to 189 with a tenfold increase in size (2 to 20 m), and from 107 to 150 with a fourfold increase in age (6 to 24 months). This suggests that the ability of Model 3 tumors to replicate the numbers of CIs seen in lung cancers ($10^{1}-10^{2}$) is robust to changes in size and age, and may be improved by increased computational resources, which may allow the simulation of larger and older tumors. We emphasize that this conclusion holds only at the tested selection strength of s=0.1 and mutation rate μ=0.4, which we chose because of their observed similarity to TRACERx tumors; size and age may have a greater effect on tumor properties when simulating different evolutionary dynamics.

This in turn suggests that the assumption of ongoing explicit competition for existing space allows us to more accurately model the underlying biology. We can confirm this by examining the relationship between selection and “clonal sweeps.”

#### 3.1.3 Clonal Sweeps Rely on Ongoing Competition Throughout Tumor Growth

We finally consider the relationship between selection strength s and $N_{truncal}$, the number of mutations fixed in all M samples and thus excluded from our main summary statistics. As discussed, these cannot be directly compared to the TRACERx cohort, as truncal mutations in real tumors may have occurred pre‐transformation (i.e., before the start of the timeframe considered by the simulations). However, patterns of truncal mutations can illustrate the emergent dynamics of simulated tumor growth.

In Model 1 (Figure 5, left panel) we observe that the number of truncal mutations is extremely low, fewer than 10 in all cases and no higher than 1 when more samples are taken (see Figures S14 and S15, topmost panels). This strongly implies that the founding cell of the tumor is generally the MRCA of all sampled tumor cells, and that no clonal sweeps occur after tumor initiation. An exome mutation rate of μ=0.4 per cell division suggests that, in cases where only one mutation is clonal (meaning that the MRCA only has one more mutation than the founding cell), that mutation appeared within a few divisions of tumor initiation; if clonal sweeps occurred later, then the MRCA would likely have accumulated more mutations during its own evolution, and those mutations would also have become clonal. Model 1 therefore produces “branching growth,” i.e. unbroken diversification from the founding cell.

We can confirm this by examining the number of mutations observed in all regions (see Figure S16), which increases with selection. Combined with the absence of clonal illusions (see the left panel of Figure 4 in the previous section), we may conclude that selection drives the emergence of fitter lineages which maintain a “universal” subclonal presence but rarely take over entire regions. In general, competition between cells in Model 1 is not sufficient to allow clonal sweeps or regional subclonal expansions: fitter lineages slowly increase in prevalence as the tumor grows, but never eliminate their less‐fit competitors. These growth patterns are laid out in Figure 6 (left panel).

Model 2, however, successfully produces tumors with clonal sweeps (see 5, middle panel). Unlike Model 1, in which no tumor ever has more than 1 truncal mutation if sufficiently sampled, the inclusion of explicit competition in Model 2 produces simulated tumors with 10–20 truncal mutations (This decreases as more regions are sampled: see Figures S12 and S13, middle panel.) This suggests that fit lineages which appear early enough in lesion growth– within the first few dozen cell divisions after transformation– can drive competing lineages to extinction and become the MRCA of the sampled tumor. After this, no mutation is able to fix across all samples, even when it is left to grow to $∼10^{8}$ cells (and $∼10^{4}$ demes); all later mutations are sample‐private. We also observe that in Model 2, selection does not increase the number of mutations seen in all regions (see Figure S16, middle panel).

Taken together, these observations suggest that explicit competition for expansion in Model 2 in fact *limits* the spread of fitter lineages, relative to Model 1 (no competition). Model 2 assumes that the tumor expands into normal tissue in “waves” when its total pressure becomes too high, with surface cells competing with each other for newly available space and resources, then this limits the speed with which mutations can spread through the tumor. A late‐arising lineage, even if it quickly outcompetes all other cells in its deme, will not be able to invade multiple sampled regions if it arises when the tumor is almost grown and has only a few “expansions” left. Almost all relevant competition occurs early, when there is still time for a lineage which outcompetes its neighbors to become the ancestor of some or all of the sampled tumor regions. This constraint acts as a ‘bottleneck’ on the effect of selection: a selection coefficient of s=0.1 is no more likely to result in a clonal sweep (see Figure 5) or clonal illusion (Figure 4, middle column) than s=0.05. We can conclude that in Model 2, growth is dominated by a small number of mutations which sweep the tumor when it is very small, after which lineages compete within samples but rarely escape their region of origin (see Figure 6, middle column).

We observe that Model 3, which assumes that cells can recolonize existing tissue, *can* produce repeated clonal sweeps. The average number of truncal mutations, $N_{truncal}$, is an order of magnitude higher than Models 1 and 2 (averages can reach up to 60 at high selection levels, when all previous averages were lower than 2), and increases with selection after s=0.02 (see Figure 5, right panel), though this increase slows at high selection levels. This rapid increase suggests that competition for existing space (Model 3) removes the bottleneck introduced by explicit competition for new space (Model 2): we have extended the “time limit” by which a fit lineage must emerge to fix in multiple samples. We conclude that the introduction of explicit competition for *existing* space in Model 3 allows the continual replacement of less‐fit clones with fitter cells. We therefore observe the late regional expansions characteristic of real lung cancers (see Figure 6, right panel). A visualization of the clonal distributions produced by all three models is provided in Figure 7.

### 3.2 Competition for Existing Space Provides the Best Approximation to the Properties of Lung Tumors

The measurements above suggest that, whilst computationally expensive, competition for existing space as in Model 3 (rather than for new space in an expanding tumor, as in Models 1 and 2) provides the best foundation (amongst the models tested) for accurately replicating the dynamics of ongoing subclonal expansion in lung cancer. A full comparison of summary statistics in all three models for M=2 is provided in Figure 8 (see Figures S17 and S18 for M=5,8). We find that, across all summary statistics, tumors from Model 3 compare better to the TRACERx cohort than those from Models 1 and 2, regardless of death rate.

We note that “fixed and private” mutations are systematically underestimated among all models and all numbers of sampled regions (though Model 3 remains the best at replicating these subclonal expansions). This attribute cannot be easily adjusted using model parameters, because changes which make it easier for a mutation to become fixed in a region (e.g., an increase in selection) will also make it easier to spread beyond that region, which is to say it will make it harder for mutations to stay *private*.

It is also likely that the detection thresholds we apply to simulated mutations, as described in Section 2.5, do not fully reflect the complexity of the filters applied to real TRACERx tumors. These depend on aspects of lung tumor biology not explicitly included here (copy number alterations, tumor purity, and so forth), and may be more conservative when determining whether a mutation is detected in multiple regions (and so more likely to classify it as “private”). It is also possible that tumor and effective deme size (i.e. the size of the neighborhood within which cells compete locally) vary between tumors, and that tumors with more fixed‐and‐private mutations tend to be larger or have smaller effective deme sizes. Our assumption that all tumors have a fixed “deme size” may be limiting our ability to replicate subclonal expansions, even as it increases the computational feasibility of the study.

Whilst we cannot currently capture the numbers of clonal illusions seen in all tumors, Model 3 nonetheless represents a significant advancement in three‐dimensional models of solid tumor evolution– specifically in its ability to replicate the regional subclonal expansions seen in NSCLC. We can now leverage this new, more accurate model to infer the selection strength of individual tumors in the TRACERx cohort.

### 3.3 Assessing Selection Strength in Tumors Using Bayesian Inference

Two‐ and three‐dimensional model frameworks are frequently used to make tumor‐specific inferences about cancer evolution (e.g., tumor age, time of metastasis seeding, fraction of cells which are stem cells, etc). We have seen in Section 3 that model assumptions about cell competition– whether competition is explicit or implicit, for new or existing space, and so on– can have profound effects on the simulated growth dynamics. These differences may have knock‐on effects on the results of inferences from real tumors. Here, we test this proposition by using the three models discussed above to infer selection strength from specific tumors in the TRACERx cohort.

Our procedure for inferring continuous selection strength s from tumor data incorporates both Approximate Bayesian Computation (ABC) and machine learning (ML). Here we take half of the cohort of simulated tumors and train a regressor (XGBoost‐Regressor) to predict s from their summary statistics. The difference between the predictions assigned to two tumors then becomes our distance function to assess the dissimilarity of those tumors. Specifically, if a real and simulated tumor are described by summary statistics $S_{i}^{real}$ and $S_{i}^{sim}$ respectively, and our regressor assigns a predicted selection strength $s_{pred}\left(S_{i}\right)$ based on a tumor's summary statistics, then we define the distance function

$$D\left(S_{i}^{real},S_{i}^{sim}\right)={\left(s_{pred}\left(S_{i}^{real}\right)-s_{pred}\left(S_{i}^{sim}\right)\right)}^{2}$$

This distance function is equivalent to the L2 distance between the *regressor's assessments* of the selection strength in the real and simulated tumors. We then choose the 1% of simulated tumors with minimal D, and examine their *true* selection strength to infer the likely selection strength in the real tumor.

Our strategy amounts to constructing a probability distribution for selection strength s from the properties of the simulated tumors which a regressor “believes” to be most similar to the real tumor (see Figure S1). We test this pipeline on simulated tumors and find that the choice of model does not affect the pipeline's ability to determine selection strength in simulated tumors (Supporting Information).

#### 3.3.1 Inferring Selection Strength in TRACERx Tumors

We can test this proposition directly by applying each inference pipeline to the cohort of TRACERx tumors (see Figure 9). As models make different assumptions about the effect of driver mutations on cell growth, the inferred selection strength cannot be directly quantitatively compared across models, in the same way that selection coefficients derived *from the same model* might be quantitatively compared. Although it is true in all models that a value of s=0.05 implies that drivers have a greater effect on cell fitness than if s=0.01. However, a value of s=0.05 derived from Model 1 will not lead to the same dynamics as a value of s=0.05 derived from Model 2.

**FIGURE 9:**
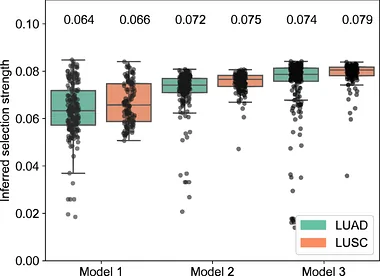
The selection strength inferred from each tumor in the TRACERx cohort using each model as a baseline. Each dot represents one tumor. For Model 1 and Model 2, we assume the maximum baseline death rate of $\lambda_{0}=0.38$. Predictions are adjusted for sample number: selection is inferred from tumors with M samples using an ABC‐ML pipeline trained on simulated tumors with M samples. Inferred selection values are averaged across 10 repeats, where each repeat uses a model trained on a different train‐test split of the simulated tumors; each point on the scatter plot represents the mean of the 10 resulting point estimates; the boxplot represents the distribution of these means.

It is precisely this model‐dependence that motivates our study. Selection coefficients are increasingly inferred from real tumors using computational models [12] in order to classify tumors as “neutral,” “low‐selection” or “high‐selection,” and such inferences may one day be used to inform treatment decisions. As a result, we seek here to establish in a cohort of real tumors how the choice of underlying model meaningfully affects the inferred effect of driver mutations.

We find that more stringent assumptions about cell competition result in higher inferred selection levels, for both LUAD and LUSC tumors. A pipeline trained on Model 2, which implements explicit competition, will infer significantly stronger selection across tumors than a pipeline trained on Model 1, which assumes that selection only infers a cell's ‘inherent’ fitness properties (Model 1 vs. Model 2, LUAD: p<0.001 [95% confidence interval on difference 0.008 ‐ 0.011]; LUSC, p<0.001 [0.007‐0.011]; all differences calculated with 1‐sample two‐sided *t*‐test; see Figure S21). Using a framework based on Model 3, which assumes cells compete for existing space in a fixed‐size tumor, results in still‐stronger inferred selection (Model 2 vs. Model 3, LUAD: p<0.001 [0.002‐0.004]; LUSC, p<0.001 [0.003‐0.005]).

This implies that our ability to infer the strength of driver mutations from real tumors is strongly influenced by model assumptions about what selection actually does. In particular, in all models, we infer slightly higher selection strength from LUSC than LUAD tumors, consistent with previous observations that LUSC tumors have more regional subclonal expansions [45].

We conclude that using Models 1 and 2, in which competition for survival is weaker and cells in existing tissue can never be replaced, will lead to the inference of lower s values (implying weaker effects of driver mutations) than when making inferences from Model 3. These poorly‐calibrated, weak‐competition models may become a “self‐fulfilling prophecy”: when models are not carefully calibrated to the underlying properties of lung cancer, and make only limited assumptions about the practical consequences of selection on cell survival, the resulting inferences may affect the inferred strength of individual driver mutations. Our full results are summarized in Table 3.

**TABLE 3:** A description of the criteria used in this paper to decide whether each model is a “good fit” to NSCLC TRACERx tumors. Here “comparable” means tumors with the same number of sampled regions.

Criterion	Type	Model 1	Model 2	Model 3
Can the model produce the median **TMB** of comparable TRACERx tumors?	Benchmark	Yes	Yes	Yes
Can the model produce the median **TMB‐priv** of comparable TRACERx tumors?	Benchmark	Yes	Yes	Yes
Can the model produce the median number of **clonal illusions** of comparable TRACERx tumors?	Benchmark	No	No	Yes
Across summary statistics, which model has the highest fraction of simulations which fall within the 5th and 95th percentiles of summary statistics for comparable TRACERx tumors?	Relative			x

## 4 Discussion and Conclusion

Over the last fifteen years, extensive genetic intra‐tumoral heterogeneity has been detected in a variety of cancers [59, 60]. This in turn has fuelled debate over the role of selection in tumor development, as researchers have attempted to determine whether this intra‐tumoral heterogeneity (ITH) reflects fitness differences between cell lineages or develops as a result of neutral diversification [32, 35, 61]. Computational models have become an increasingly popular tool for investigating the rules which govern tumor growth [5, 11, 12, 34], and in particular for distinguishing between the presence and absence of genetic selection [8, 10]. However, recent research has made it clear that tailored models are necessary to capture the properties of different tumor types and thus make reliable cancer‐type‐specific inferences [9].

In this work, we sought to build a model capable of replicating the clonal sweeps characteristic of NSCLC tumors. We hypothesized that this would require stringent and explicit cell‐cell competition for space and resources. Many agent‐based models of competition [8, 46, 47, 48] keep computational costs low by treating the base unit of the simulation as a “group” of cells, which elides cell‐level mutational processes. In order to generate simulated multi‐region whole‐exome sequencing data (for comparison with a real‐world lung tumor cohort), we built models in which cells were represented individually. We began with a model in which cells competed implicitly for space and resources, based on SCIMET [11], with proliferation and death assumed to be an inherent property of a cell's genotype and space allocated to fast‐growing population. We chose this model as our starting point because it has previously been used to draw inferences about modes of evolution from a variety of cancers, including lung cancer [12]. We then imposed increasingly strict forms of competition, with cell fitness depending explicitly on the genotypes of cells in their immediate neighborhood (“active competition” [49]). Our best‐performing model (amongst those tested) encodes explicit multi‐level (cell–cell and region‐region) competition, which to our knowledge has not been included in previous SABMs of lung tumor evolution. Whilst direct comparison between increasingly complex models is complicated by differences in final tumor size, we believe that explicit competition mechanics should be considered when building future models of lung cancer, especially for those used to identify selection pressures in the clinic. They should also be considered when modeling subclonal expansions in other solid cancers (for example, those observed in the breast [62]).

These results also have implications for attempts to identify and predict the lineages which drive metastatic spread [57]. Recent analyses of single‐cell NSCLC data have suggested that subclones which have higher potential to seed metastases are also those which *divide* more [63]. Our findings imply that this enhanced proliferation may be linked to invasiveness, in that these fitter cells both divide more *and* kill off their neighbors to spread through existing regions of a tumor. This invasiveness would enhance the ability of highly proliferative clones to colonize normal tissue and seed metastatic lesions. This link may also partly explain why some forms of genomic ITH have been linked to poorer patient prognosis [45]. Further experimental work is necessary to confirm these findings and investigate their full implications.

We also sought to quantify the impact of model design choices on our ability to infer selection strength from real tumors in the TRACERx cohort, using Approximate Bayesian Computation and machine learning. We found that, when using an inference pipeline based on this “two‐stage, explicit competition” model, we inferred significantly stronger selection across the cohort (in both LUAD and LUSC tumors) than when using a simpler “implicit competition” model. This suggests that the assumptions of tumor evolution models may partly be “self‐fulfilling”: models which assume very little explicit competition between cells may lead us to infer a more limited effect of driver mutations on cell fitness. We also note that this analysis is made possible only by multi‐region sequencing data (which allows us to identify clonal illusions), as the tumor‐mutational burden alone is insufficient to separate model performance.

As with all modeling‐based analyses of tumor growth, our study is limited by computational power and a lack of reliable estimates for some parameters. In our model, computational costs are kept low by discarding information about cells in the interior of the tumor (which are assumed to die and are never sampled), as well as a novel ancestry‐tracing algorithm which only assigns mutations to cells at the end of the simulation, and then only for sampled cells. Whilst this keeps the simulation lightweight and allows more explicit forms of cell–cell competition than are considered in the existing literature, it makes continuous tracing of lineages through the tumor functionally impossible. In addition, our ability to distinguish neutral and high selection in Model 3 (the explicit‐competition model) is likely dependent on our assumptions about the age of the tumor (1 year) and the size of the local environment (10 000 cells). While these assumptions are clinically reasonable, they are also determined by computational constraints, which prevent us from conducting full sensitivity analyses for all fixed parameters. Generating tens of thousands of simulations of much larger, older or more spatially granular tumors is not currently computationally feasible. We also decreased final tumor size as model complexity increased, which may affect comparisons between models.

To prioritize the ability of our modeling framework to represent individual cells and generate multi‐region sequencing data, we abstracted or elided other aspects of realistic tumor biology, which may impact our conclusions. These simplifying assumptions include: a uniform effect of driver mutations (i.e., a single global selection strength s, between 0 and 0.1); a uniform mutation rate μ=0.4 exome mutations per division; neutral passenger mutations; a spatio‐temporally uniform death rate (for Models 1 and 2, where an explicit death rate $\lambda_{0}$ is encoded); a uniform deme size $N_{0}$; and proliferation/death only within “active” surface demes. We abstract all mutations into “point” mutations and do not explicitly model copy‐number or structural variations. The model did not include explicit representation of nutrients or mechanical pressure, instead mediating competition by limiting the number of cells which can exist in any one deme. We also do not model interactions with normal or immune cells. Our optimization processes also rely on computing genetic information only at the end of the simulation from ancestry records, and then only for cells which are included in the final bulk tumor samples. This means that genetic information for all cells in the tumor is not kept continually accessible. Any attempt to compute ancestry for all cells at each timestep would increase computational expense by several orders of magnitude; similarly, to keep a growing list of all mutations possessed by each living cell would also lead to infeasible RAM requirements. This precludes continual tracking of the spatial progression of specific clones over the course of the tumor's evolution. This is a significant limitation of the modeling framework, but a necessary consequence of the optimization processes needed to represent cell‐competition dynamics in the level of detail we include here. Importantly, this precludes easy visualization of the clonal dynamics within the three‐dimensional model, as tracking clonal spread across the tumor would require a full ancestral reconstruction of all visualized cells. Future work should focus on developing modeling frameworks which balance ease‐of‐visualization with computational efficiency, in order to facilitate the intuitive understanding required for clinical adoption.

Amongst the model classes tested, the best‐performing model we identified is still only able to produce numbers of clonal illusions on the lower end of the appropriate range. Our models also tend to overestimate the number of mutations shared between regions, suggesting that overall we are still underestimating the full extent of regional diversification in lung tumors. We note that our conclusions are based on the assumption of spatial uniformity. Models which incorporate angiogenesis, or other spatial variations in division or death rate, are likely to see faster clonal sweeps: clones which reach more proliferative environments (due to random spatial variation, or proximity to nutrient‐dense blood vessels) will expand quickly and have a lasting advantage. Similarly, variations in the size of the local environment (i.e., deme size $N_{0}$) will allow fit clones to expand more quickly when they find themselves in larger demes, increasing the overall ability of fit cells to encounter and outcompete less‐fit cells. Further work should incorporate these realistic aspects of tumor biology, perhaps by leveraging greater computational power than was available here to increase tumor age or include competition with normal cells (as in the work of Noble and coworkers [9]).

This work demonstrates the mechanistic insights that can be gleaned from tumor‐type‐specific models of cancer evolution. Future simulation studies should carefully examine their hard‐coded assumptions about inter‐cellular interactions, and benchmark the properties of their model tumors against real data. This work establishes a precedent for tumor‐type‐specific inference models, and should motivate researchers to conduct these benchmarks as a matter of routine. This is a vital first step to the incorporation of computational models into clinical practice.

## Conflicts of Interest

N.M. holds European patents relating to targeting neoantigens (PCT/EP2016/059401), identifying patient response to immune checkpoint blockade (PCT/EP2016/071471), determining HLA LOH (PCT/GB2018/052004), predicting survival rates of patients with cancer (PCT/GB2020/050221). J.F. is a member of DAiNA scientific advisory board.

## Supporting information


**Supporting File**: advs76764‐sup‐0001‐SuppMat.pdf.

## Data Availability

The data that support the findings of this study are openly available at https://github.com/hcoggan/Agent‐based‐simulations‐of‐lung‐tumour‐evolution.
