# Supplementary material for: Agent‐Based Simulations of Lung Tumor Evolution Suggest That Ongoing Cell Competition Drives Realistic Clonal Expansions
Source: Adv Sci (Weinh). 2026 Aug 6;13(58):e76764. doi: 10.1002/advs.76764 (PMC13445327; doi:10.1002/advs.76764)
Supplement: Supplementary file 1 [file ADVS-13-e76764-s001.pdf]

Agent-based simulations of lung tumor evolution suggest that ongoing cell competition drives realistic clonal expansions: supplementary material

Helena Coggan, James R. M. Black, Carlos Martínez-Ruiz, Kristiana Grigoriadis, Jasmin Fisher and Nicholas McGranahan

May 2026

The document details computational methods relevant to the work described in this paper, and contains figures supplementary to the main analysis.

1 Model design

Here we provide full details of individual models presented in the main text. The full code for each model is available in the Github hcoggan/Agent-based-simulations-of-lung-tumour-evolution (version tag march-2026).

1.1 Defining Model 1: The 'indirect competition' model

Our first model assumes that selection affects only a cell's likelihood of division, and that cells compete only indirectly for space and resources. Within demes, cells have no impact on each other's outcomes (division or survival). We use this model to test whether the properties of lung cancer, in particular recent subclonal expansions, can be replicated in a model that assumes fitness is an inherent property of a cell. The model can be thought of as an adaptation of SCIMET [1; 2; 3] with non-uniform mitotic age and saturating selection effects.

1.1.1 Division and death

As in all models, driver mutations increase a cell's probability of division asymptotically towards 1. Cells die with a fixed baseline probability λ_0 per timestep; this death rate is a variable simulation parameter. Cells with more driver mutations tend to die less, so that a cell with k driver mutations (with selection strength s) dies with a per-timestep probability

$$\lambda(s,k) = \lambda_0(1-s)^k$$

Cell death does not occur if demes are at 10% capacity or less (i.e. $N \leq 0.1N_0$). As tumor growth is driven by full demes splitting in half (see below in Section 1.1.2), these 'mostly empty' demes are extremely unlikely except in the very early stages of tumor growth, when only one deme is occupied. We impose this rule to avoid the stochastic death of all tumor cells when the tumor is still very small. Mechanistically, this represents saturating competition with the environment: cells in a largely empty deme have enough resources to divide without dying until the population breaches some threshold (here 1000 cells). Division and death are both probabilistic, which ensures variable mitotic age: in any given timestep a cell need neither divide nor die.

1.1.2 Cell migration and tumor growth

When a deme becomes full (i.e. $N > N_0$ after division and death), its population splits in half and colonizes a randomly-chosen empty neighboring lattice-point, if one exists. This creates an implicit inter-deme competition for space, with fitter cells dividing faster and colonizing space that less-fit cells might otherwise have used. Less fit cells are likelier to be trapped in the center of the tumor and are less likely to be sampled at the end of the simulation. At every timestep, demes are iterated over in a random order, with cells dividing and dying probabilistically and demes splitting where they are over capacity. This has the effect of randomly breaking ties between equally-fit demes competing for the same empty neighboring lattice-point at the same timestep.

1.1.3 Computational constraints

The relatively simple assumptions about competition in this model mean that it is able to achieve a final tumor size of roughly 500 million cells. The time taken to reach this size varies, depending on the baseline death rate λ_0. The average fractional growth rate ϵ of the founding lineage is given by $1+\epsilon = (1+p_0)(1-\lambda_0)$. As division and death are both stochastic, setting ϵ too close to zero results in tumors that stall or shrink, and never reach full size; experience suggests that a minimum growth rate of around $\epsilon = 0.01$ is necessary to avoid this. This corresponds to $\lambda_0 = 0.38$, which we use as our maximum death rate. We explore death rates $\lambda_0 = [0.0, 0.1, 0.2, 0.3, 0.35, 0.38]$, which will result in tumor ages of between forty days and five years. (These calculations are based on the properties of the founding cell, and will necessarily be overestimates if driver mutations sweep the tumor and increase the overall growth rate; we take them as a guideline here.)

1.2 Defining Model 2: The 'direct competition, continual-expansion' model

In the second model, we assume that cells compete both within and between demes. Competition between cells is for survival, so that a cell's probability of dying is no longer dependent only on its own genotype but on its surrounding ecology. Demes compete based on their relative fitness to move into empty

space, instead of expanding only when they are full. The tumor continually expands, and tissue that has been colonized can never be *recolonized*. We use this model to test whether the properties of lung cancer can be replicated by a model that assumes that a cell's fitness is relative within a continually expanding tumor, i.e. that there is no competition for existing space.

1.2.1 Division and death

A cell's proliferative fitness (i.e. its probability of dividing per timestep) depends on its driver mutations exactly as in 1.1.1.

Here a cell also has a 'survival-fitness', given by $f_s = (1+s)^k$. This increases without bound with selection strength s and number of driver mutations k, as it is only ever measured relative to the fitness of other cells. When a deme is at more than 10% capacity, cells within it compete for survival: this encodes the idea that, in an environment with limited resources, cells are less likely to survive if there are a large number of fit cells in their vicinity. For a baseline death rate of λ_0, a cell's probability of dying in a given timestep is given by

$$\lambda = \max(1 - (1 - \lambda_0)\frac{f_s}{\bar{f}_s}, 0)$$

where $\bar{f}_s$ represents the average fitness of all cells in the deme. Cells die with probability λ_0 if their fitness is equal to the average fitness of cells in their local environment; a fitter cell $(f_s > \bar{f}_s)$ is more likely to survive. This eliminates the possibility of true 'asymptotic selection', as survival probabilities are now interdependent. In Model 1, as cells gain driver mutations, their probabilities of both survival and proliferation increase asymptotically towards 1 at every timestep, and there will come a point at which further driver events 'stop mattering': if all cells in a deme have some large number k of drivers, a cell with $k+1$ drivers is no longer meaningfully advantaged. In Model 2, survival probabilities are calculated from *relative* fitnesses, so the emergence of a cell with an extra driver will force up the death rates of all other cells and decrease its own, regardless of the baseline number of drivers k. This avoids a scenario in which selection pressures eventually 'die off', and evolution becomes neutral: in Model 2, cells can always get fitter.

Cells divide and die until their demes are over-capacity $(N > N_0)$ at the end of a timestep, at which point both processes stop until the next 'splitting event', described below.

1.2.2 Cell migration and tumor growth

If cells compete for survival, the population of a deme is no longer a reasonable proxy for its average population fitness. In Model 1, fitter cells divide more and die less, so if one deme reaches capacity before another, we can reasonably say that the cells within that deme have 'outcompeted' their neighbors. A 'first-come, first-served' approach to deme splitting, with over-capacity demes immediately claiming nearby empty space, therefore represents implicit competition for space and resources that fitter cells would be expected to win.

In Model 2, competition for survival decouples the relationship between the overall death rate in a deme and the fitness of its cells. As fit lineages emerge, they increase the likelihood that their less-fit neighbors will die, which might cause the population of the deme to drop even as its average fitness rapidly increases. As a result, we cannot allow demes to split when they become full if we want 'fitness' within the simulation to have any meaning at all (i.e. if we maintain that increasing fitness should increase the likelihood that a cell's descendants will be sampled from the tumor surface). We therefore assume that demes split *all at once*, when the tumor as whole reaches a threshold cell density and runs out of space to contain its growing population. (We could alternatively assume that demes split individually when they become a certain amount fitter than their immediate neighbors, but this would prevent growth in neutral tumors.)

We implement these splitting events when all active demes (those with empty neighbors) are over-capacity. The 'splitting algorithm' works as follows:

1. Identify all empty demes which neighbor *at least one* full deme.
2. Iterate over those empty demes in a random order. For each empty deme:
   (a) Identify all full demes neighboring this deme. If there are none remaining, move on to the next empty deme.
   (b) If there are full neighboring demes, choose one to split into the empty deme. The probability of choosing deme i, where the cells have average survival-fitness $\bar{f}_s^i$, is $\frac{\bar{f}_s^i}{\sum_j \bar{f}_s^j}$, where the sum over j in the denominator encompasses all demes competing for the empty deme. This is to say that demes compete for space and resources. Each deme has a chance of success linearly proportional to its relative fitness, reflecting the dynamics of competition between cells. Of course, if there is only one full deme trying to expand, then this will be chosen regardless of its fitness.
   (c) Move each cell from the full deme into the empty deme with probability ½.
3. If a deme is still full at the end of this simulation, mark it as 'inactive' and stop tracking cells within it. All empty demes identified in step 1 are either no longer empty or have no remaining full neighboring demes. Thus, any deme still *full* must have been outcompeted and cannot have any empty neighbors remaining, and so has no chance of splitting at a future timestep. The cells within it will not be sequenced at the end of the simulation, when samples are taken from the tumor's surface.

After the splitting event, division and death resumes. This continues until the tumor reaches full size.

1.2.3 Computational constraints

Competition both between cells and between demes requires repeated comparisons at every timestep, which increases the computational demands of this

model. As a result, we are limited to a final tumor size of 100 million cells. We explore death rates $\lambda_0 = [0.0, 0.1, 0.2, 0.3, 0.35, 0.38]$ as in Model 1.

1.3 Defining Model 3: The 'two-stage, direct competition' model

In our final model we assume that cells compete both within demes (for survival) and between demes (for both empty and occupied space). Tumor evolution proceeds in two stages: one 'exponential' and one 'homeostatic'. This is an idealization of the general principle that growth slows as the tumor size increases, and that cells in a large tumor compete more fiercely for space and resources.

In the first 'exponential' stage, cells divide without dying, and demes split when full. At the first timestep at which the population of the tumor exceeds 5 million cells, tumor growth stops. We assume during the second 'homeostatic' stage that the tumor has run out of space to grow into, and that an increase in local cell density triggers competition, not expansion. Cells spread through the tumor by out-competing their less-fit neighbors for space and nutrients; cells which lose such competitions die, and yield their resources to fitter, invading lineages.

We use this model to test whether allowing cells to invade occupied demes and replace less fit lineages is sufficient to reproduce the properties of lung cancer. This two-stage approach is adapted from the work of Zhao and coworkers [4], who assumed that cells in colorectal cancer glands continue to age (i.e. divide and die) after the maximum number of glands has been reached, but has not been deployed in any three-dimensional model of cancer growth to our knowledge.

1.3.1 Division and death

In all cases, cells proliferate as described in Section 1.1.1. During the first stage of growth, there is no cell death. During the second, demes which are over capacity $(N > N_0)$ after the division step are forced to compete for survival, as it is assumed they have run out of space. As in Model 2, a cell with k drivers of strength s has survival-fitness $f_s = (1+s)^k$. A cell with survival-fitness f_s survives with probability $p = \min(1, \frac{N_0 f_s}{F})$, where F is the total fitness of all cells in the deme, and dies otherwise. If the deme contains N cells of equal fitness, then their probability of survival will each be N_0/N, and we will expect roughly N_0 cells to survive at the end of the step. Thus, cells in a deme compete on fitness for sufficient space and resources. Over time, fitter lineages should slowly take over the population of a deme. This assumption means the simulation has no independent death rate parameter λ_0.

1.3.2 Cell migration and tumor growth

During the first stage of tumor growth, demes split into empty neighboring demes when full, as there is no competition and so fitter cells merely proliferate faster. After a split, both demes are then under-capacity and proliferation can resume.

This expansion continues until the tumor's total population exceeds 5 million cells. (This stage of tumor growth can be thought of as a smaller version of Model 1 with no cell death, such that $\lambda_0 = 0$.)

In the second stage, in order to allow fitter lineages to spread beyond the demes they arise in, we also implement competition *between* demes. At the end of each timestep, the populations of fitter demes split in half (i.e. each cell moves with probability 0.5 and stays otherwise) and replace the populations of their less-fit neighbors. We assume that all cells in the invaded deme die and disintegrate immediately, so that space and nutrients are available to the cells replacing them.

The process of replacement is probabilistic. If deme i is a neighbor of deme j, and the average survival-fitnesses of their populations (before the replacement step) are $\bar{f}_s^i$ and $\bar{f}_s^j$ respectively, then the probability that deme j will be become available to be replaced by deme i is

$$p(i \to j) = \begin{cases} 1 - \frac{\bar{f}_s^j}{\bar{f}_s^i} & \text{if } \bar{f}_s^i \geq \bar{f}_s^j \\ 0 & \text{if } \bar{f}_s^i < \bar{f}_s^j \end{cases}$$

This is a parameter-free function designed to ensure that less-fit demes will never replace fitter demes, and that replacement-availability becomes certain $(p(i \to j) \to 1)$ as $\frac{\bar{f}_s^j}{\bar{f}_s^i} \to 0$, i.e. as deme i becomes infinitely fitter than deme j. If deme i has several demes available to be replaced by it in a single timestep, then it chooses one of these neighbors at random. Each deme can only be replaced, or replace another deme, once in a timestep, though a deme that has invaded another may itself be invaded by a fitter deme later in the same timestep. As in the expansion stage, demes are iterated over in a random order, so that ties between two demes competing to expand into the same less-fit neighboring deme are broken at random.

A deme which splits randomly in half to replace a neighboring deme should not see any changes in its average fitness; a deme which is replaced should see its fitness increase. This means that the process of replacement is inherently self-limiting. Once a deme has replaced its neighbor, the two populations should have similar fitnesses, and so neither will be likely to replace the other until a fitness differential has built up between them over many timesteps of separate evolution.

As before, proliferation only occurs in demes with at least one empty neighboring lattice-point. The second stage of this simulation therefore takes place entirely on the tumor surface. Surrounded demes are assumed to be low in nutrients and high in mechanical pressure, so living cells do not compete to invade them. Lineages arising during this stage of evolution can colonize the entire tumor by spreading through surface demes.

1.3.3 Computational constraints

The process of replacement means that tumors continue to evolve whilst being kept at a fixed size. This breaks the traditional link between size and age in computational models of tumor evolution [2; 3; 4; 5; 6], and allows us to simulate

the dynamics of cell competition in a fixed-size tumor. However, this stage of evolution is very expensive, and scales more than linearly with the number of cells in this tumor. To keep our model computationally feasible, we set the fixed size to be 5 million cells.

The model proceeds for 365 timesteps, or one simulated year. By this point, roughly 1 billion cells should have been produced (assuming 5 million cells undergoing 0.631 divisions per day for around 300 days), though only a fraction survive.

1.4 Sampling tumors

At the end of the simulation, we take 8 simulated samples (the maximum number obtained from any tumor in the TRACERx dataset [7]), each comprising 1% of the tumor's surface cells (i.e. 1% of active demes). We ensure that samples are as distant from each other as possible by randomly generating 20,000 possible combinations of 8 central demes, calculating the sum of the total pairwise distances between them, and choosing the set of demes which are furthest apart according to this metric.

Once 8 spatially-separated demes have been chosen, we generate samples from the surrounding tissue. We treat these demes as the 'central points' of the sample, and take the 1% of demes closest to them (roughly 5 demes per sample for the smallest Model-3 tumors, and many more for Models 1 or 2). We check that none of these samples are overlapping (i.e. we have not chosen the same deme twice). If we have, we repeat the selection procedure described above, as many times as necessary, until we have 8 spatially-separated non-overlapping samples. If the population of any sample N_i exceeds $N_c = 50000$ cells, we include each cell in the sample with probability N_c/N_i, resulting in a sample of roughly $N_i' \approx N_c$ cells. This approximates the size of samples from tumors in the TRACERx cohort [8].

1.5 Realistic sequencing of simulated tumors

We assume a tumor purity of 80% and a total read depth of 400x, translating into a mean read depth of $R =$ 320x. We further assume that our sequencing protocol is unable to detect mutations in a sample if their CCF in that sample is less than 1%. In the TRACERx dataset, real tumors use a threshold which begins at 0.05% and increases as tumor purity decreases. We choose the more stringent end of this threshold largely for computational efficiency, to avoid simulating the sequencing of many low-frequency mutations which will end up being filtered out by the region-specific region thresholds described later in this section.

We assume an error rate of $\epsilon = 0.0001$ (0.01%) inherent to the sequencing process. Thus a mutation with identifier j, with true CCF $p_{ij} \geq 0.01$ in sample i, has a detected CCF

$$\tilde{p_{ij}} = \frac{R_{ij}}{R_{eff}}$$

where R_{ij} is the number of reads of mutation j in sample i, distributed binomially as $R_{ij} \sim Bin(R_{eff}, p_{ij}(1-\epsilon))$, and R_{eff} is the effective read depth (see below). We further assume that, at this sequencing depth, there is minimal variation in the number of reads at each locus, such that we can assume a uniform read depth. Since we do not model copy number alterations, we assume all regions of the genome have a copy number of 2. Somatic mutations are heterozygous, and so a sequencing depth of $R = 320$x means that, on average, each locus in the genome is read 160 times. We therefore set the effective read depth $R_{eff} = 0.5R = 160$x, so that we have 160 'attempts' to read each mutation. This allows us to avoid further increasing the amount of stochasticity in the model.

We recognize that these assumptions may affect the distribution of observed mutational CCFs in simulated tumors. To minimize the dependence of our model results on the precise shape of this distribution, we choose summary statistics which depend only on whether a mutation is *fixed* (CCF=1.0), *unfixed* (0 $<$ CCF $<$ 1.0) or *absent* (CCF=0.0) in a tumor region.

To calculate summary statistics from M simulated samples, we must first ensure these samples are well-separated. The first sample is chosen at random (out of the available 8 samples); the second sample is chosen to maximize its distance from the first; the third sample (if $M > 2$) is chosen to maximize the sum of its distances from the first two, and so on.

Once a set of samples has been chosen, we filter mutations in accordance with TRACERx stringency protocols: mutations which have fewer than 10 reads in all individual samples are excluded (i.e. to pass this step, mutations must have a detected CCF of 6.25% in at least 1 sample).

2 Inferring selection strength from real and simulated tumors

We use a variant on Approximate Bayesian Computation [9] to infer selection strength. ABC has been used by Graham and coworkers to distinguish the presence of zero, one or two selected subclones from single tumor regions [5] and multi-region samples of colorectal cancer [10]; the same team of researchers have used ABC to retrieve selection levels and time of subclone emergence from simulated multi-region tumor data [11]. Curtis and colleagues have also used ABC to deduce differentiation patterns from primary CRCs [4; 6; 12] and seeding times from paired primary and metastatic samples [2]. If fitting models to a relatively small number of tumors, researchers can use 'sequential Monte-Carlo' ABC (ABC-SMC), in which simulations are continually generated from a decreasing parameter space until they converge on the properties of a real 'target' tumor. Depending on the complexity of the simulation and the identifiability of its parameters, one can also select between models using this method, and fit as many parameters as desired (though each new parameter will increase the dimensionality of the problem, so this will depend on computational constraints).

However, ABC-SMC is necessarily iterative and difficult to parallelize (since we must evaluate the properties of some simulated tumors before deciding

which parameters to use to generate the next), and its cost scales with the number of target tumors. Here we have more than 300 evaluable TRACERx tumors, and three complex and highly stochastic simulation frameworks, which limits the dimensionality of our inference problem (ideally we would infer 1-3 parameters). We take the approach of Hu *et. al.* [2] and generate a large set of simulated tumors (tens of thousands) from a uniform prior distribution. For each target tumor, we identify the set of these simulations which most closely represent that tumor's properties, and use their parameters to produce a posterior distribution. In the main text, we seek to infer only the selection strength s.

In this work we combine both approaches, using a method developed by Jiang and coworkers [13] (Figure 1). We split the cohort of simulations in half, into a training set and testing set. We use the training set to train a regressor (here XGB-Regressor) to predict s from a the summary statistics S_i of simulated tumors. We apply this regressor to the real tumor to predict its selection strength, $s'_{pred}(S'_i)$. We then use our test set to construct a probability distribution for the selection strength. The distance of a simulated tumor with summary statistics S_i from the real tumor is given by $D(S_i, S'_i) = (s_{pred}(S_i) - s'_{pred}(S'_i))^2$, i.e. the L2 distance between the *classifier's assessments* of the selection strength in the real and simulated tumors. We then choose the 1% of simulated scenarios which are closest to the real tumor. Our strategy amounts to using ABC with the ML-driven distance function $D(S_i, S'_i)$, and thus constructing our probability distribution from the properties of the 1% of simulated tumors which a classifier 'thinks' are closest to the real tumor. This allows all the flexibility of ML whilst retaining the benefits of ABC (uncertainty estimation and constrained outputs). Our inference process is described in Figure 1).

3 Benchmarking inference on simulated tumors

Before applying our inference process to real tumors, we first benchmark it on simulated tumors. This allows us to assess how well we can infer selection strength 'in theory', because in simulated tumors, the underlying selection strength is known. Each benchmark is carried out on tumors from the appropriate model: we attempt to infer selection in Model-1 tumors using a classifier trained on Model-1 tumors, and so on. In this way we can compare how accurately selection can be inferred from each model, *assuming that the model assumptions are appropriate to the tumor*.

To keep this comparison fair, we discard simulations from Model 1 and Model 2 with death rates $\lambda_0 < 0.38$; we have seen above that simulations with low cell turnover are unable to replicate the mutational burden of lung cancer. This means that we have roughly 20,000 simulations per model, of which 10,000 are used to train the classifier; the choice of the 1% most appropriate simulations thus amounts to choosing the 100 'best' model tumors to replicate each 'target' tumor. We assess each model using leave-one-out analysis: we exclude each simulated tumor in turn from the test set and treat it as the target tumor, attempting to infer its underlying selection using the remainder of the test cohort. Because we know the *true* selection value used to generate it, this allows us to

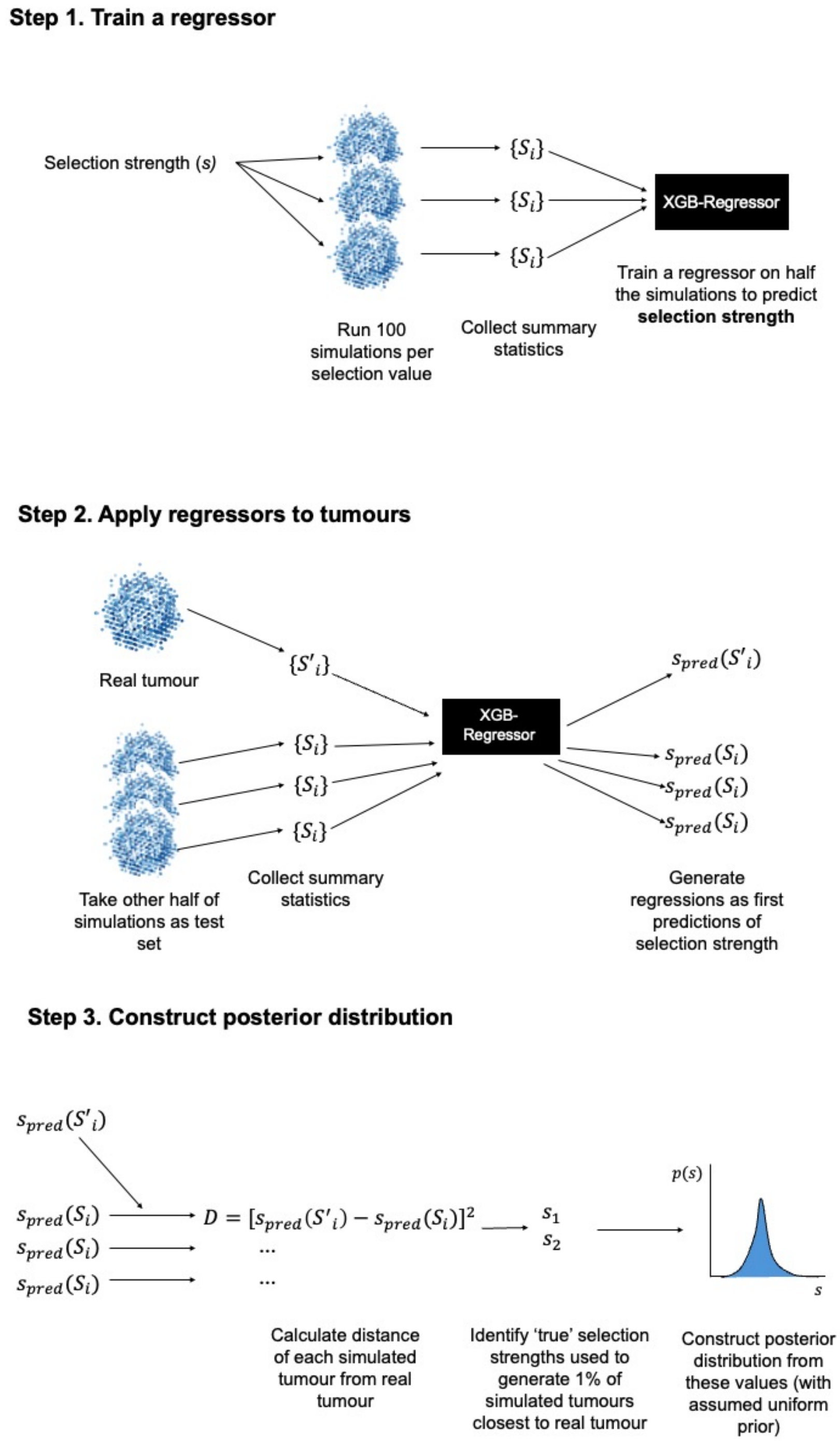

Supplementary Figure 1: A schematic of the inference process used to fit continuous selection strength to a real tumor from the TRACERx cohort. Note that the fitting process itself does not involve direct comparison of models, but instead aims to infer the selection strength that would result *if one had already chosen a particular model*.

assess the accuracy of the pipeline.

We compare the performance of this inference pipeline across models (top to bottom) in Figure 2. We can assess performance by looking both at the average MSE per prediction (i.e. the mean squared difference between true and inferred selection), and the correlation between true and inferred selection (Pearson's R). Perfect inference will result in an MSE of zero and an R of one.

We find that the inference pipeline performs similarly across all models on simulated data. MSE is lowest (and R highest) in Model 3, which best replicates the properties of TRACERx tumors; this is closely followed by Model 1, in which fitter cells divide more and die less without directly competing. Model 2 is the most difficult framework under which to infer selection, as the effects of selection are saturating; the properties of a Model-2 tumor with $s = 0.06$ are not meaningfully different from those with $s = 0.1$.

Under all models, neutral selection remains difficult to correctly identify: generally most models at $s < 0.01$ are assessed at $s \approx 0.01$. Selection is generally overestimated in the range $0.02 \leq s \leq 0.07$, and underestimated thereafter. Within Model 1 and Model 3, we see a clear peak in TMB at $s = 0.02$; inferred selection increases sharply around this point, suggesting a reliance on TMB to deduce s. We examine more directly later (Figure 3). All models show saturating inference: small differences become increasingly difficult to discern at high selection levels, such that tumors at $s = 0.1$ are usually inferred to have selection levels of $s = 0.07 - 0.08$. For all models, inference becomes increasingly accurate with more samples (see Appendix Figure 20).

This benchmarking process provides a 'best-case scenario' for the quality of inference from real tumors, assuming the inference pipeline is trained on simulations which accurately capture the properties of its target data (here, *in silico* tumors from the same model). As we have seen, these model tumors vary in their resemblance to real lung cancers, and so our ability to deduce selection strength from real tumors will be limited by how well we have modelled the underlying biology. In particular, our choice of model may influence what the classifier 'thinks of' as the properties of high-selection tumors.

Whilst selection is more or less equally easy to infer from all models, the 'decision-making process' learned by the pipeline varies significantly between models. This can be seen by examining the relative importance of each summary statistic to each regressor (as measured by the normalized gain-type importance; see Figure 19). For example, the decision-making process of regressors trained on Model 1 is strongly dependent on the number of samples, M. Selection is assigned to low-sample tumors largely based on the overall TMB (Figure 3); when more samples are present, the regressor begins to prioritize the number of mutations fixed in one sample (see Appendix, Figure 19). Regressors trained on Model 2 always make decisions based on the overall number of mutations detected, whereas those based on Model 3 focus on mutations fixed in multiple samples. These differences suggest that the choice of underlying model strongly influences the inference pipeline's idea of what a neutral or high-selection tumor 'looks like', and this in turn may bias our results when selection strength is inferred from a real tumor.

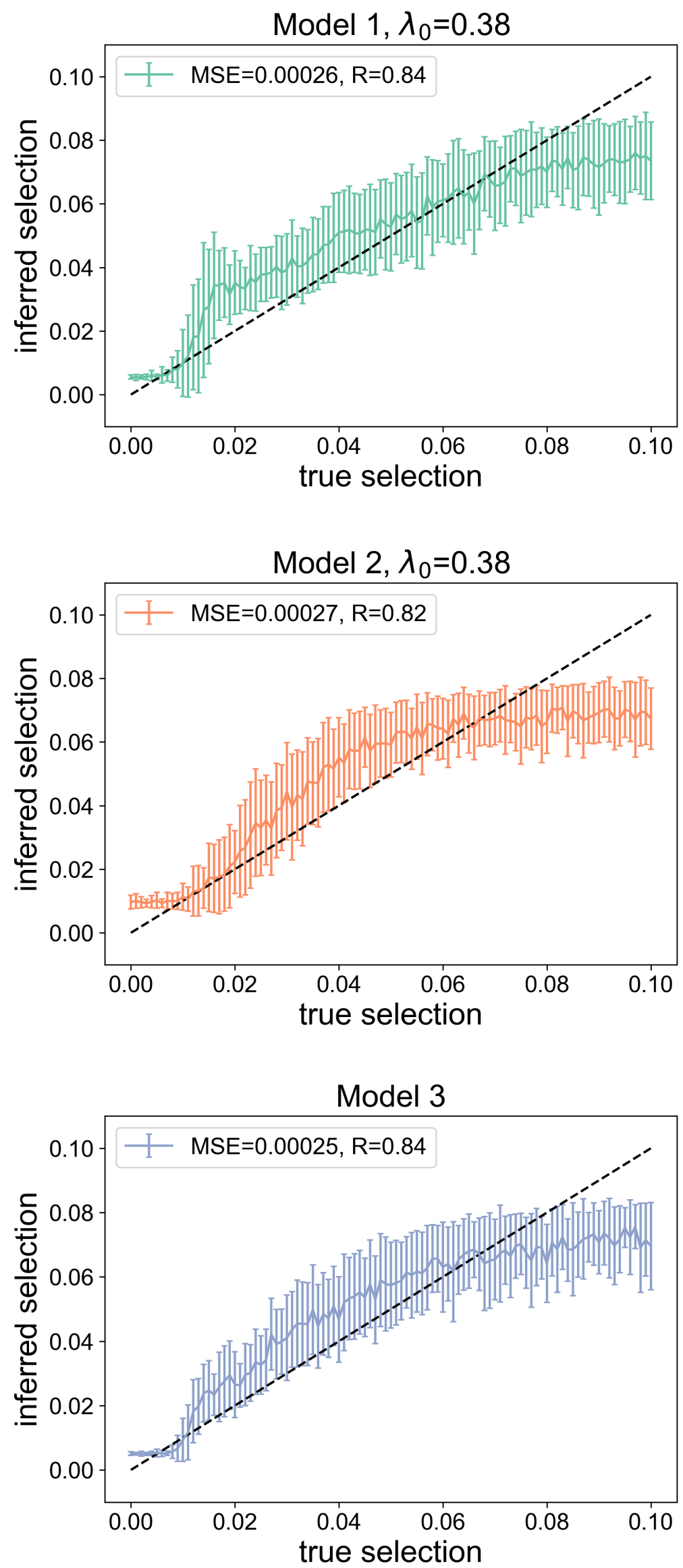

Supplementary Figure 2: The relationship between real and inferred selection for simulated tumors in Model 1 (top), Model 2 (centre) and Model 3 (bottom), assuming $M = 2$ samples. Models 1 and 2 have an assumed death rate of $\lambda_0 = 0.38$. The inferred selection is taken as the average of the selection strengths corresponding to the 1% closest tested simulations; error bars indicate the standard deviations of these inferences. The legend indicates MSE (average mean squared error across all predictions) and R (Pearson's correlation between true and inferred selection). The line y=x (corresponding to perfect inference) is plotted in black for comparison. The objective of XGB-Regressor is to minimize the MSE.

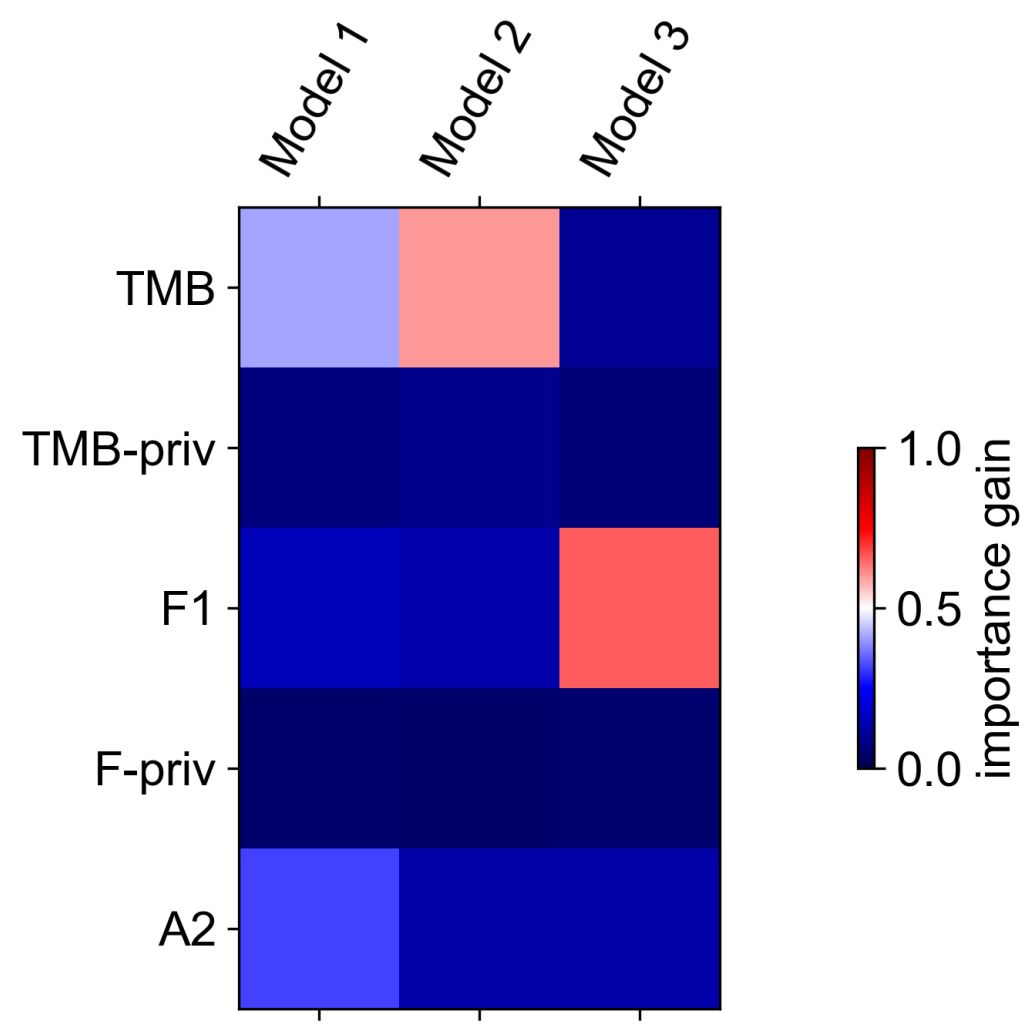

Supplementary Figure 3: The relative importance gain for classifiers trained on each of three models, assuming $M = 2$ samples. The (i, j)th element represents the relative importance gain of summary statistic i to the regressor trained on *in silico* tumors from Model j. Models 1 and 2 have an assumed death rate of $\lambda_0 = 0.38$. Importance gains are normalized across all summary statistics.

4 Pre-processing of TRACERx lung tumor data

TRACERx data is taken from the work by Frankell *et. al.* [7]. To ensure that variations in mutational frequency reflect *intra-tumor* heterogeneity, the dataset excludes samples which initially appeared to have been taken from the same tumor but were later discovered to comprise genomically distinct tumors in the same patient. Tumors have a minimum of 2 and a maximum of 8 samples (see Figure 4). The dataset comprises 382 tumors with a median of 3 sampled regions (inter-quartile range 2.0-4.0).

Mutation CCF values were computed for all mutations detected in TRACERx patients. Mutations were processed as in the TRACERx421 study [7]. CONIPHER [14] was run to compute the cancer cell fraction (CCF) of mutations and to construct mutational clusters. In cases where mutations were removed by CONIPHER during clustering, the mutational CCF in each region was computed by the following formula:

```
compute_mut_ccf <- function(vaf, purity, tum_cn, normal_cn)
{ vaf/purity * (purity * tum_cn + (1 - purity) * normal_cn) }
```

If a mutation is identified as ‘clonal’ in a particular region by the clustering algorithm used to deconvolve CCF from copy number (CONIPHER, see [14]), we round its CCF to 1.0; if a mutation has a recorded CCF greater than 1.0, we round this down to 1.0. For the purposes of comparison with simulations, we exclude all mutations which are fixed (CCF=1.0) in all regions.

When evaluating model performance (see below), we compare simulated tumors to real NSCLC tumors in the TRACERx cohort. Here we use only primary tumors with 2 or more samples, taken from patients in which only a single

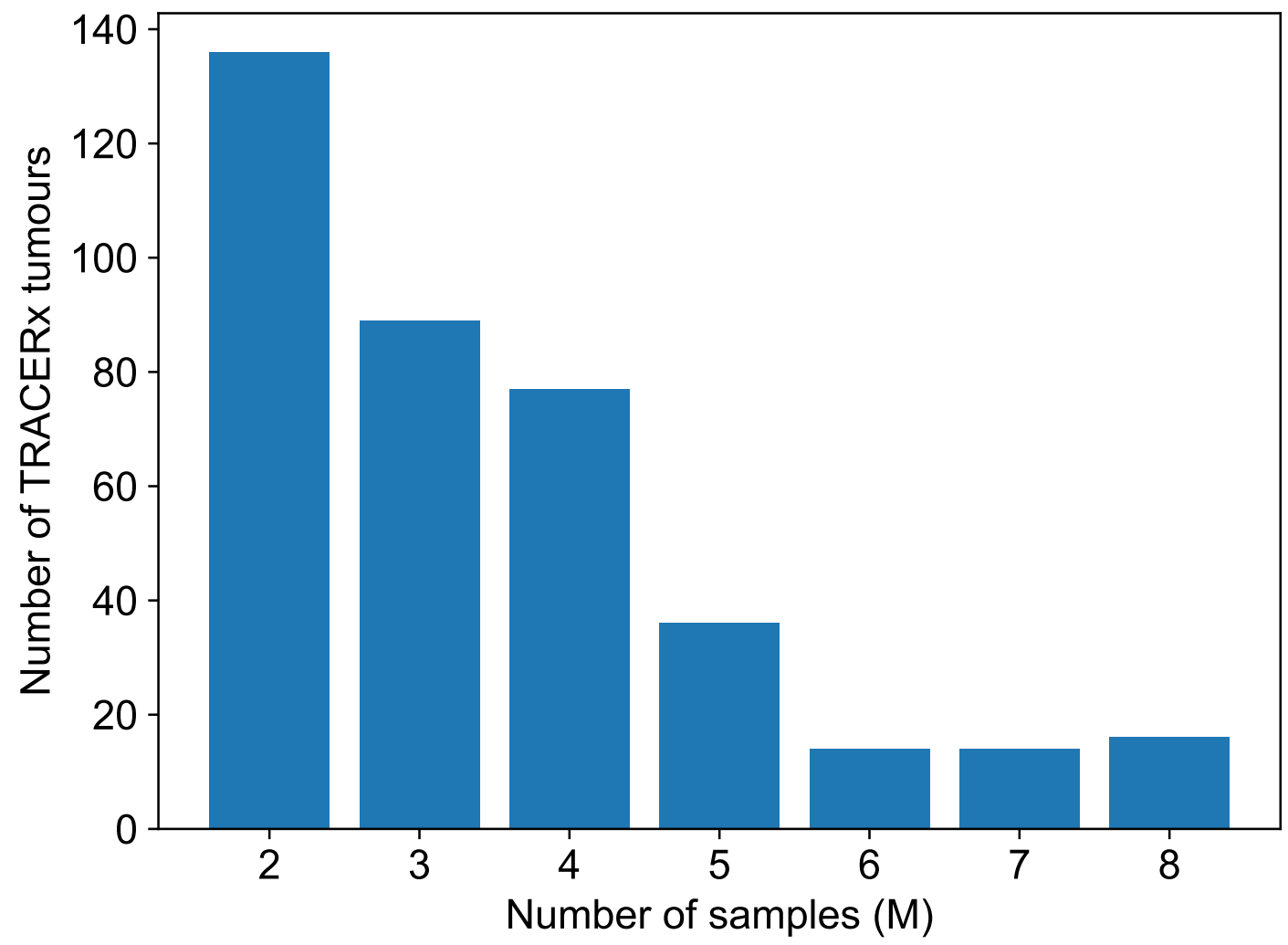

Supplementary Figure 4: A bar chart describing the number of samples taken from tumors in the TRACERx cohort.

primary tumor was found (i.e. excluding cases in which multiple genomically distinct primary tumors were identified in the same patient). We include all mutations which are trusted by the pass filter (approximately those with 4 reads in the sample of interest and 10 in at least one sample). We exclude mutations which marked as occurring 'early' in tumor evolution (i.e. occurred truncally and before whole-genome doubling WGD, an event that is known to happen early in the evolution of lung tumors [7]).

5 Supplementary Figures and Tables

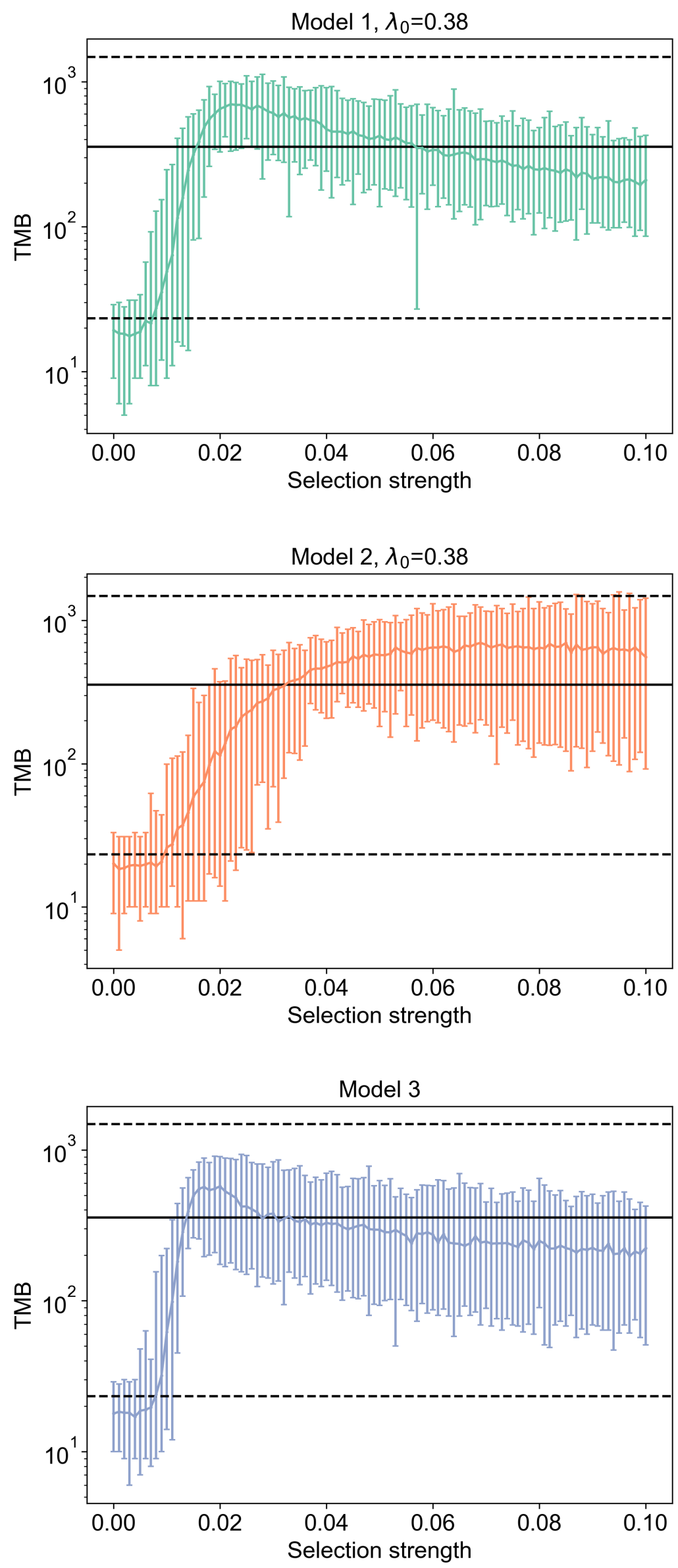

Supplementary Figure 5: The variation of simulated TMB in each model with selection strength s. Models 1 and 2 have an assumed death rate of $\lambda_0 = 0.38$. We assume $M = 5$ samples. Averages are taken from 100 repeats at each (s, λ_0) pair, with maxima and minima indicated by error bars. Dotted black lines indicate the outer limits (5th and 95th percentiles) of corresponding summary statistics from all TRACERx tumors which were sampled at M regions. Solid black lines indicate the medians of these real TRACERx tumor cohorts.

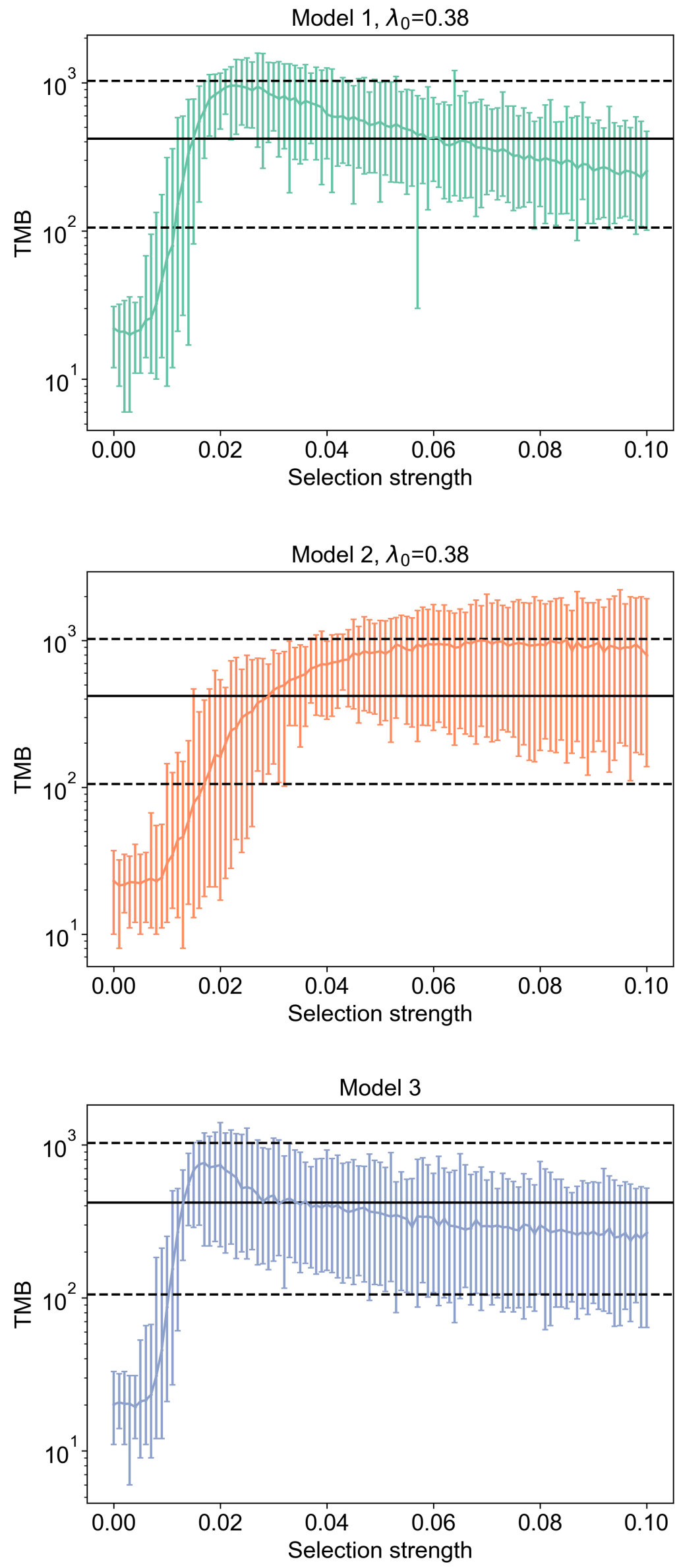

Supplementary Figure 6: The variation of simulated TMB in each model with selection strength s. Models 1 and 2 have an assumed death rate of $\lambda_0 = 0.38$. We assume $M = 8$ samples.

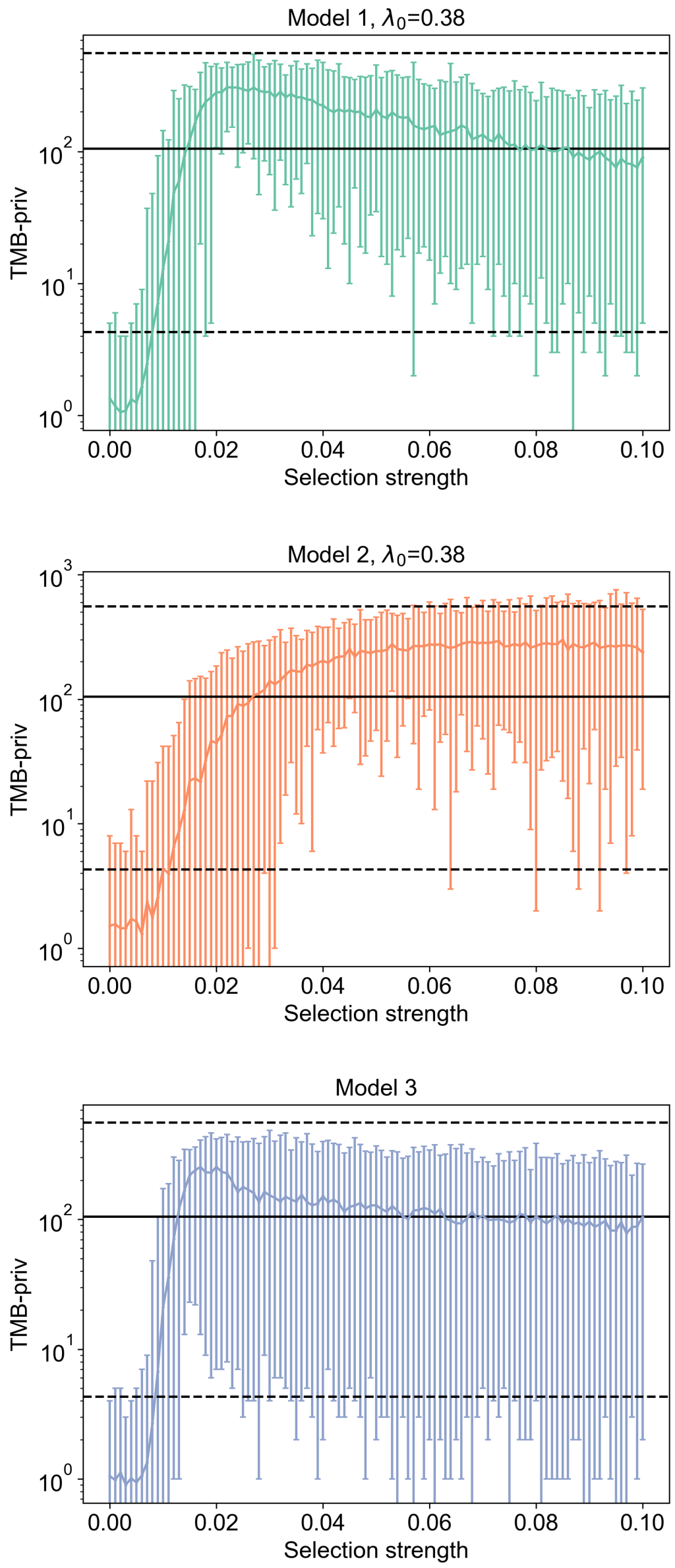

Supplementary Figure 7: The variation of simulated *private* TMB (number of mutations only detected in one region) with selection strength s. Models 1 and 2 have an assumed death rate of $\lambda_0 = 0.38$. We assume $M = 2$ samples.

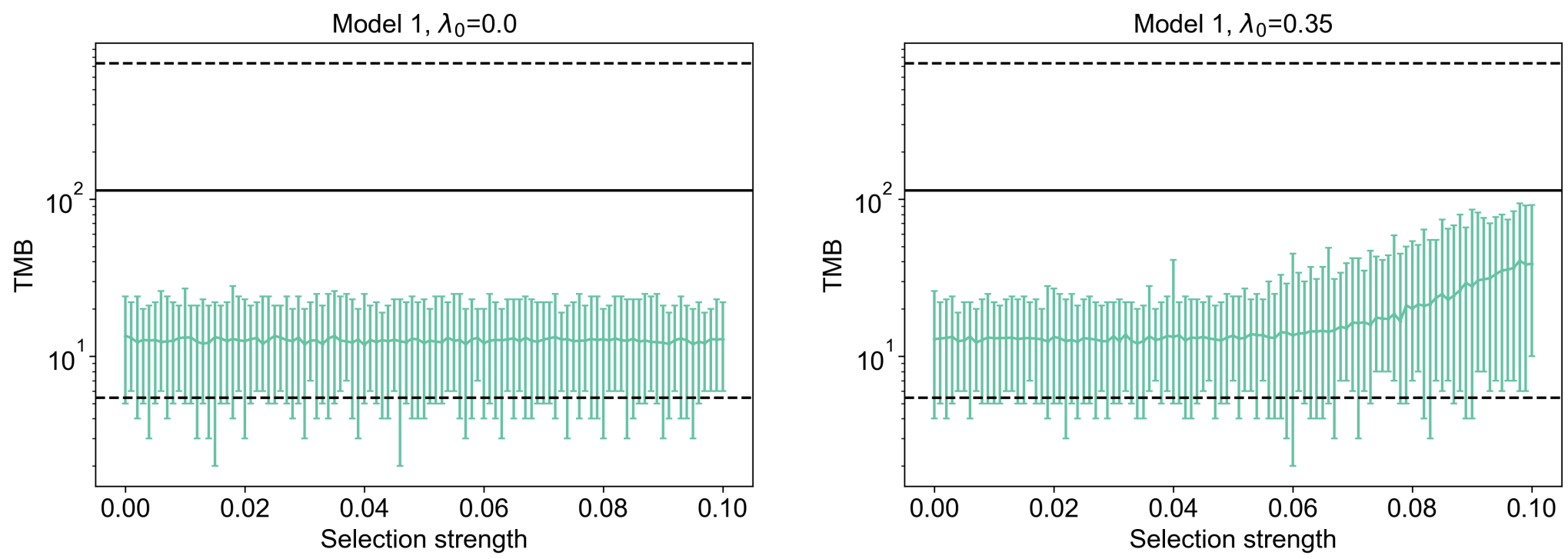

Supplementary Figure 8: The variation of simulated TMB in each model with selection strength s in Model 1, assuming a death rate of $\lambda_0 = 0.0$ (left) or $\lambda_0 = 0.35$ (right). We assume $M = 2$ samples.

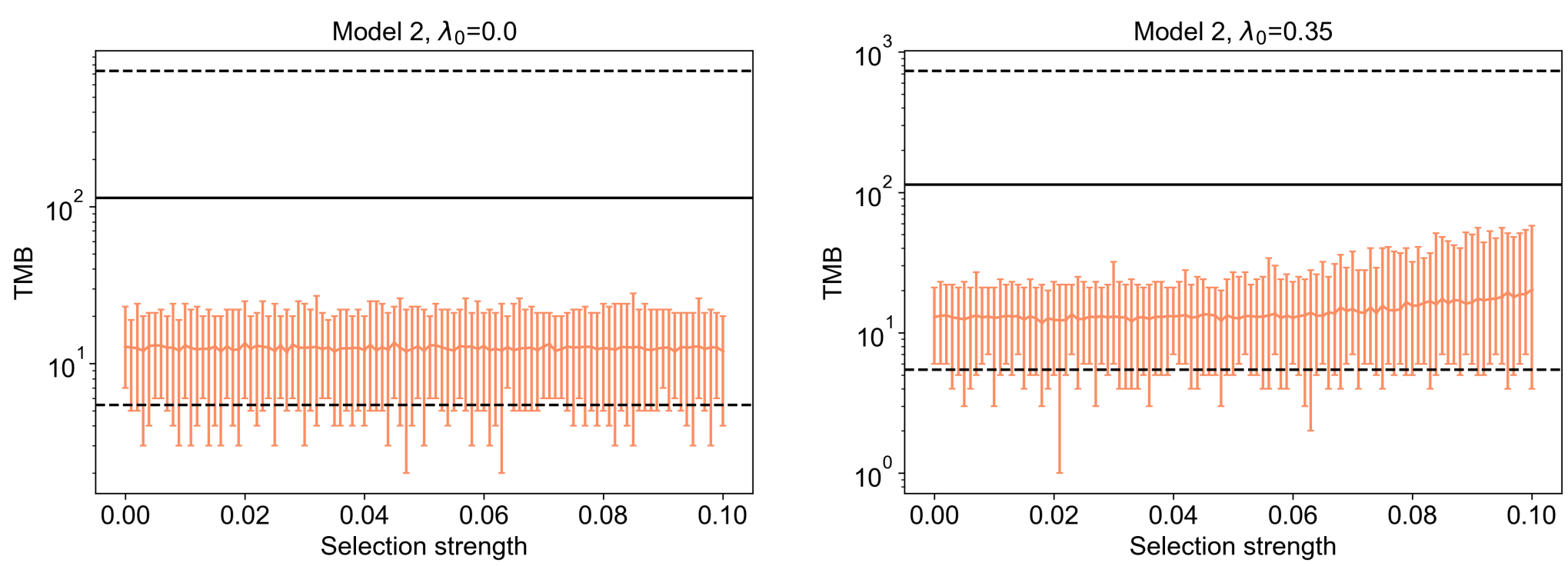

Supplementary Figure 9: The variation of simulated TMB in each model with selection strength s in Model 2, assuming a death rate of $\lambda_0 = 0.0$ (left) or $\lambda_0 = 0.35$ (right). We assume $M = 2$ samples.

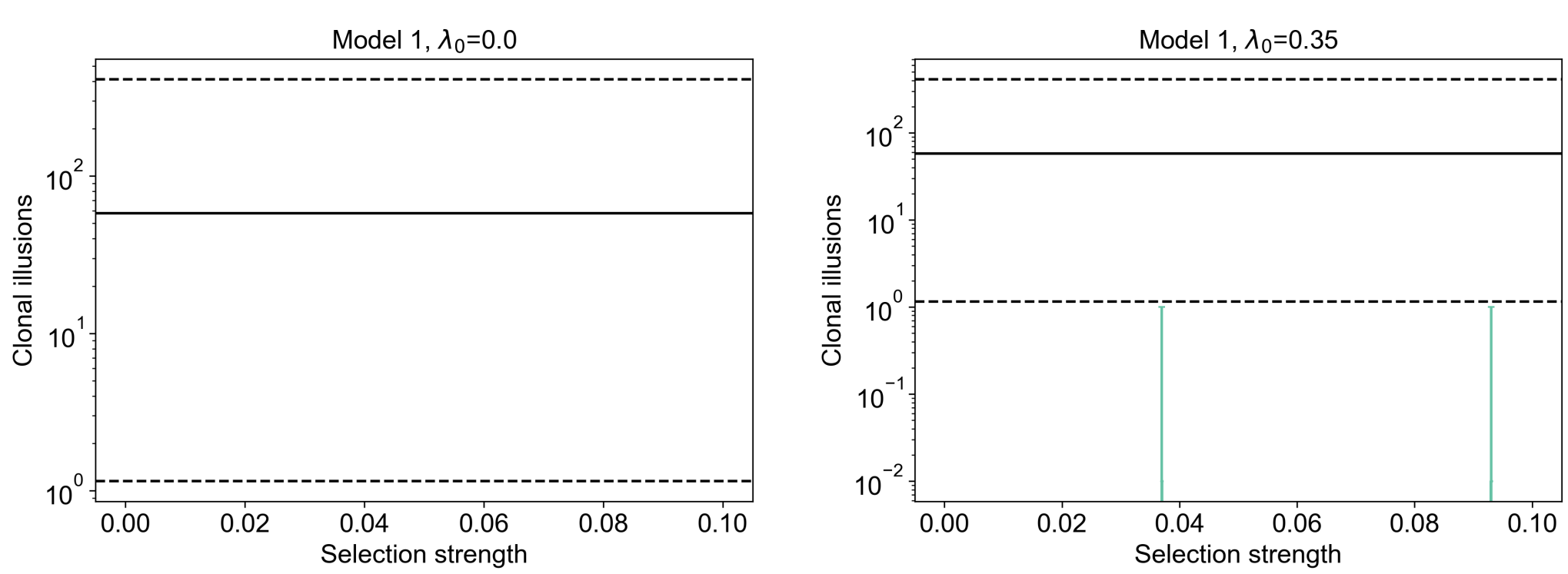

Supplementary Figure 10: The variation of simulated CIs in each model with selection strength s in Model 1, assuming a death rate of $\lambda_0 = 0.0$ (left) or $\lambda_0 = 0.35$ (right). We assume $M = 2$ samples.

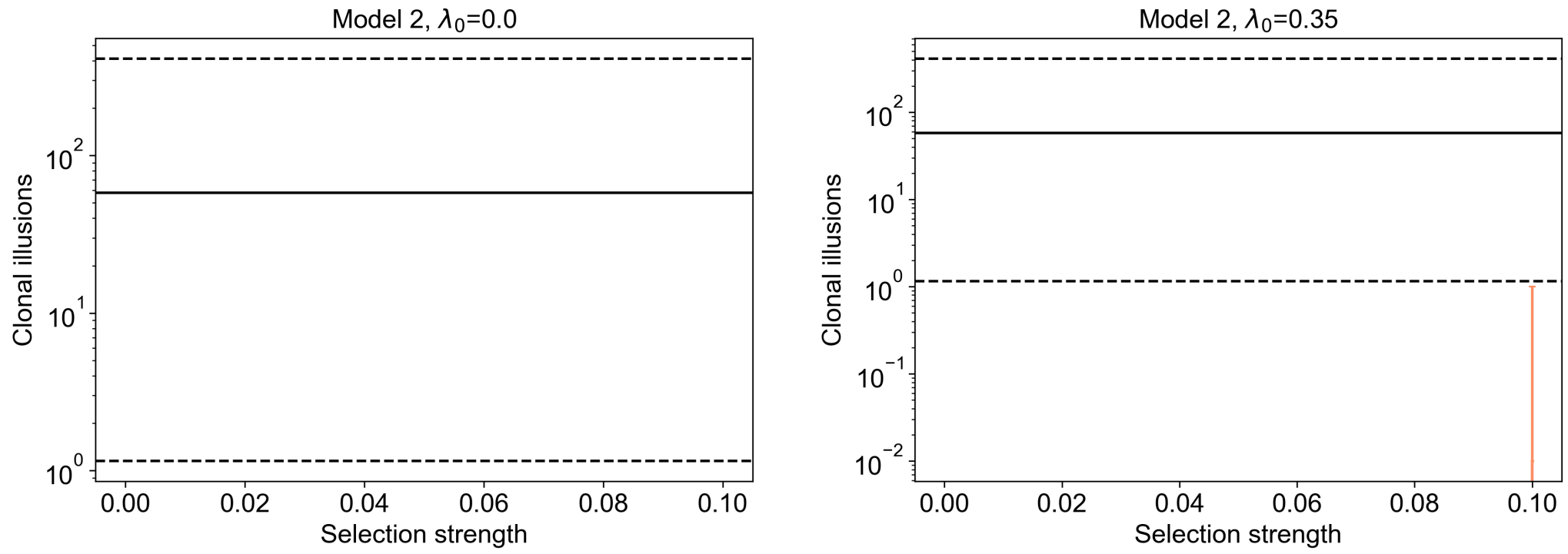

Supplementary Figure 11: The variation of simulated CIs in each model with selection strength s in Model 2, assuming a death rate of $\lambda_0 = 0.0$ (left) or $\lambda_0 = 0.35$ (right). We assume $M = 2$ samples.

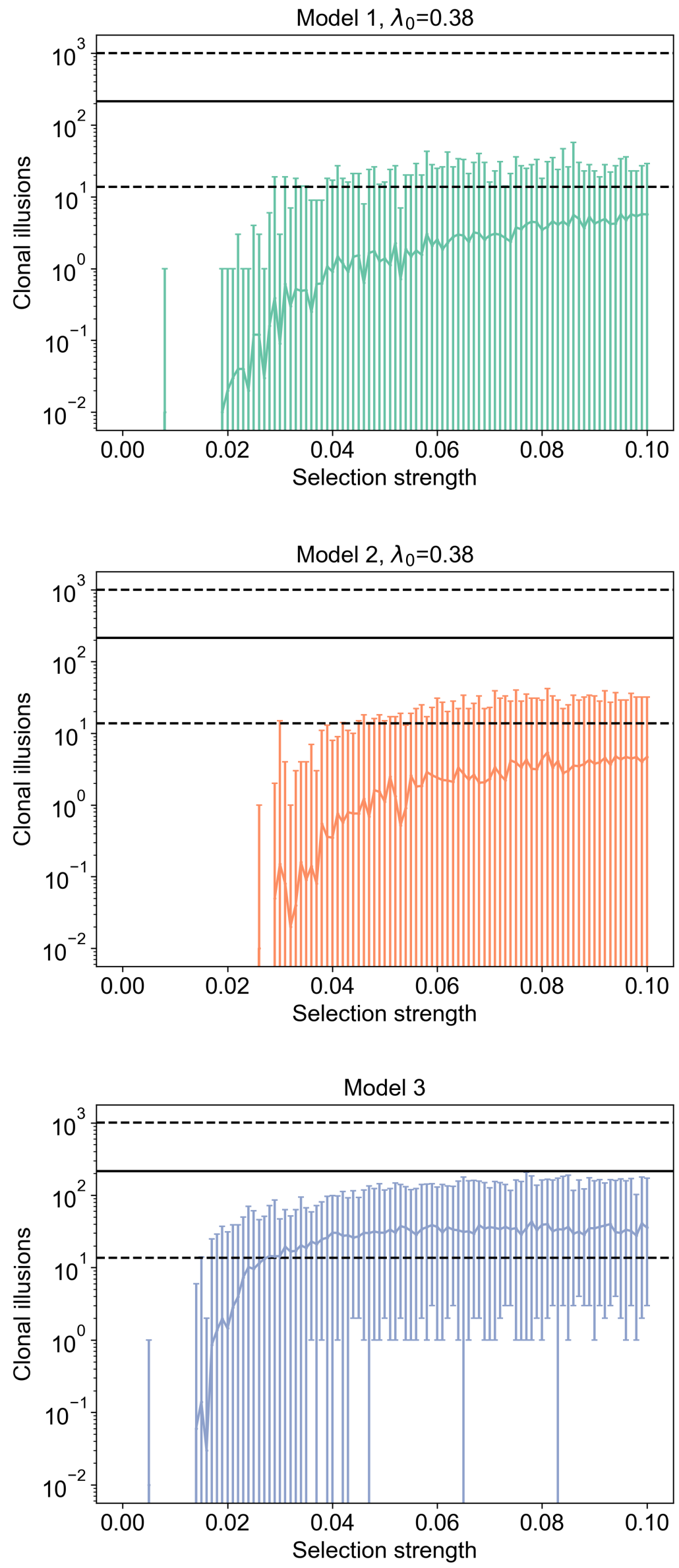

Supplementary Figure 12: The variation of simulated clonal illusions (CIs) in each model with selection strength s. Models 1 and 2 have an assumed death rate of $\lambda_0 = 0.38$. We assume $M = 5$ samples.

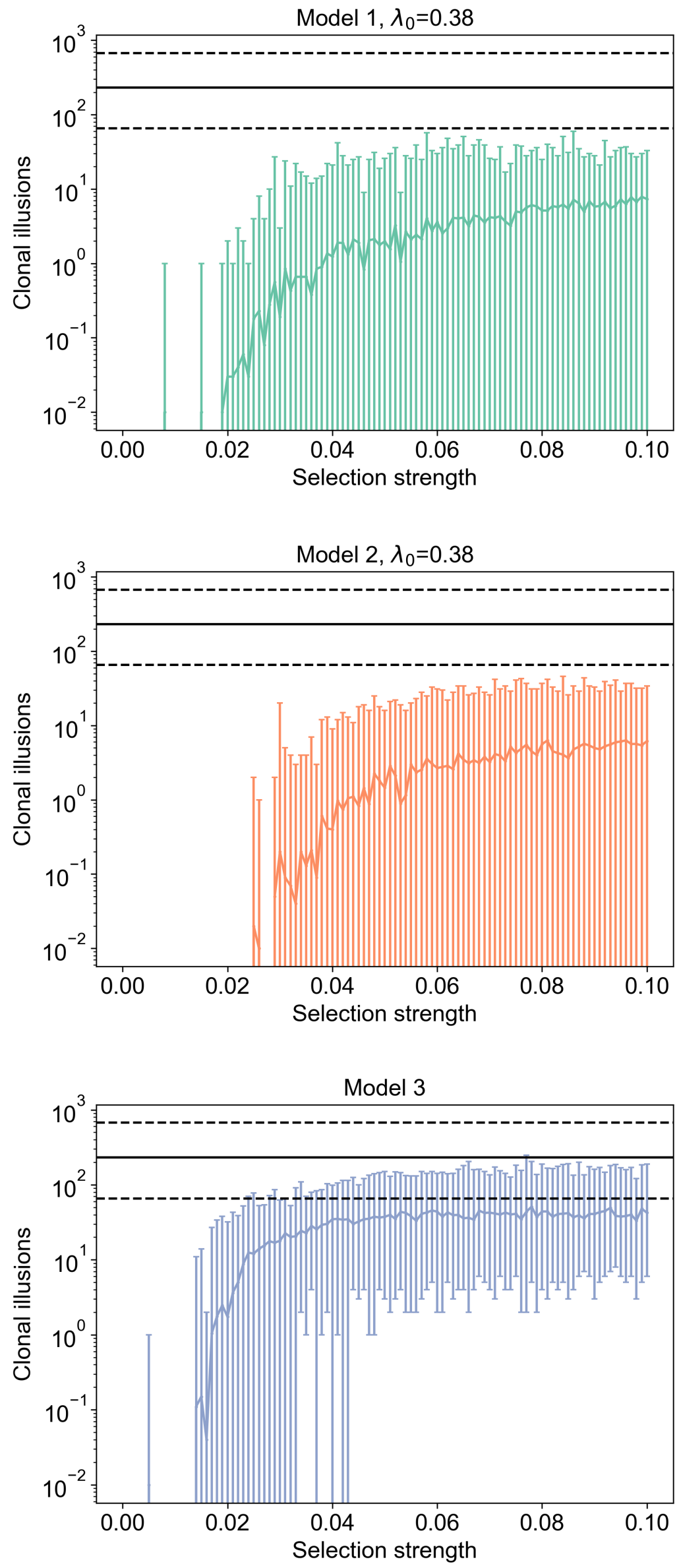

Supplementary Figure 13: The variation of simulated clonal illusions (CIs) in each model with selection strength s. Models 1 and 2 have an assumed death rate of $\lambda_0 = 0.38$. We assume $M = 8$ samples.

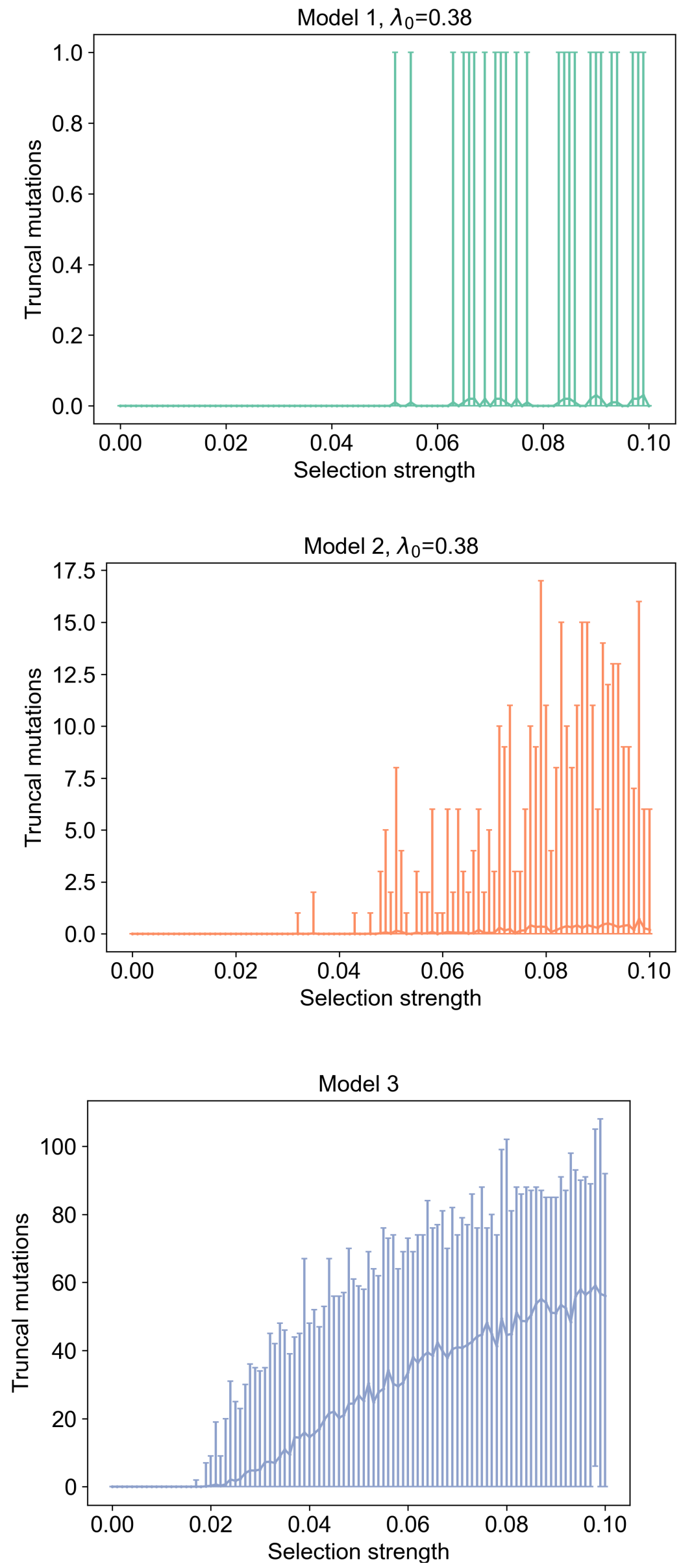

Supplementary Figure 14: The variation of the number of truncal mutations in each model with selection strength s. Models 1 and 2 have an assumed death rate of $\lambda_0 = 0.38$. We assume $M = 5$ samples.

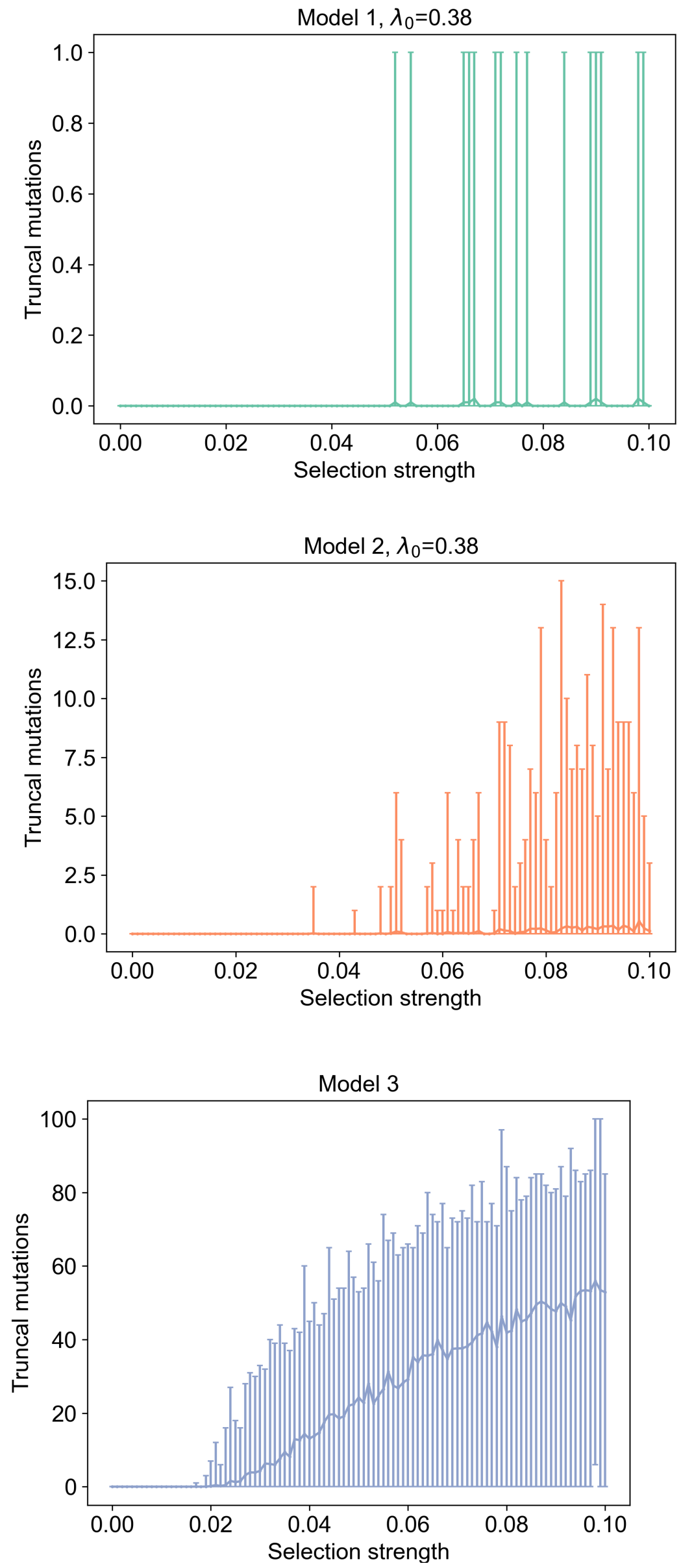

Supplementary Figure 15: The variation of the number of truncal mutations in each model with selection strength s. Models 1 and 2 have an assumed death rate of $\lambda_0 = 0.38$. We assume $M = 8$ samples.

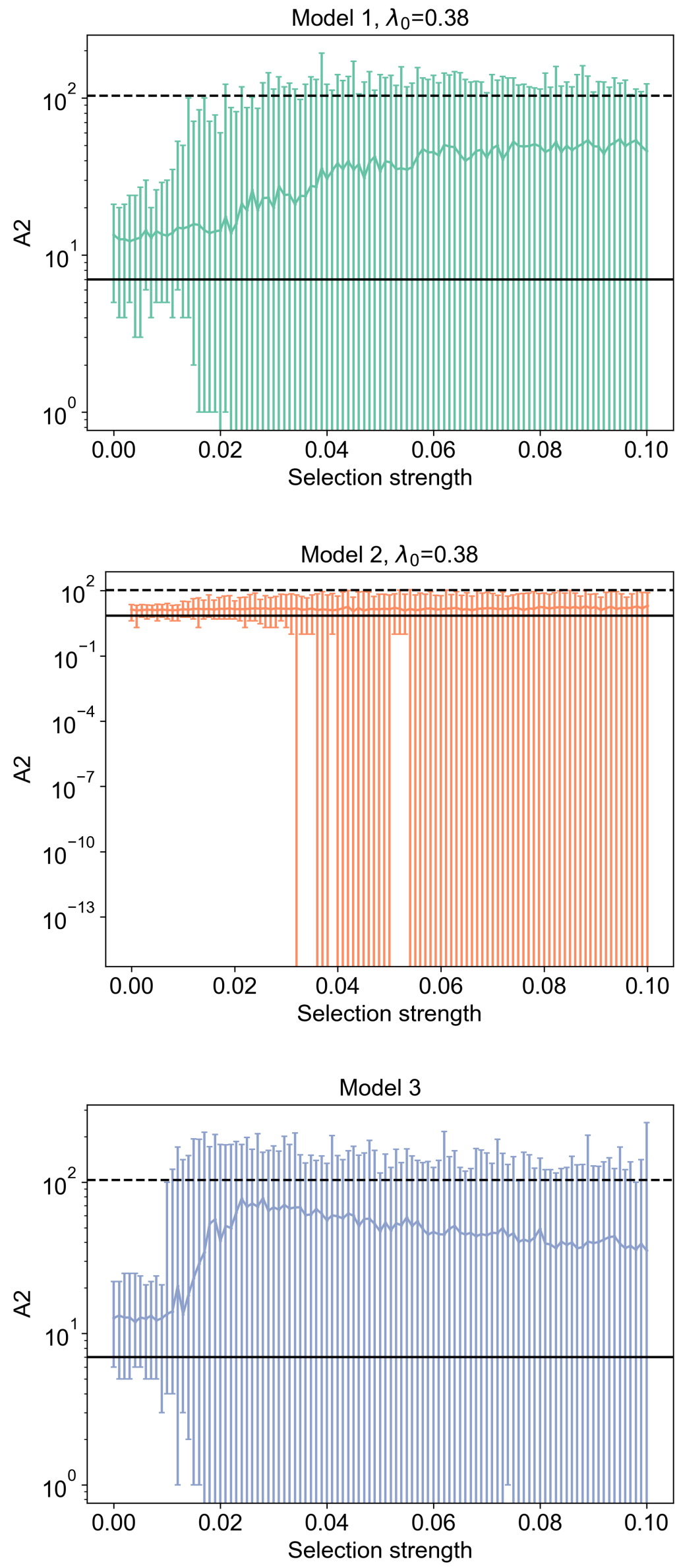

Supplementary Figure 16: The variation of the number of mutations detected in all samples (A2) with selection strength s. Models 1 and 2 have an assumed death rate of $\lambda_0 = 0.38$. We assume $M = 2$ samples. Here, the absence of a lower dotted line indicates the minimum number of such mutations in tumors from the TRACERx cohort is 0.

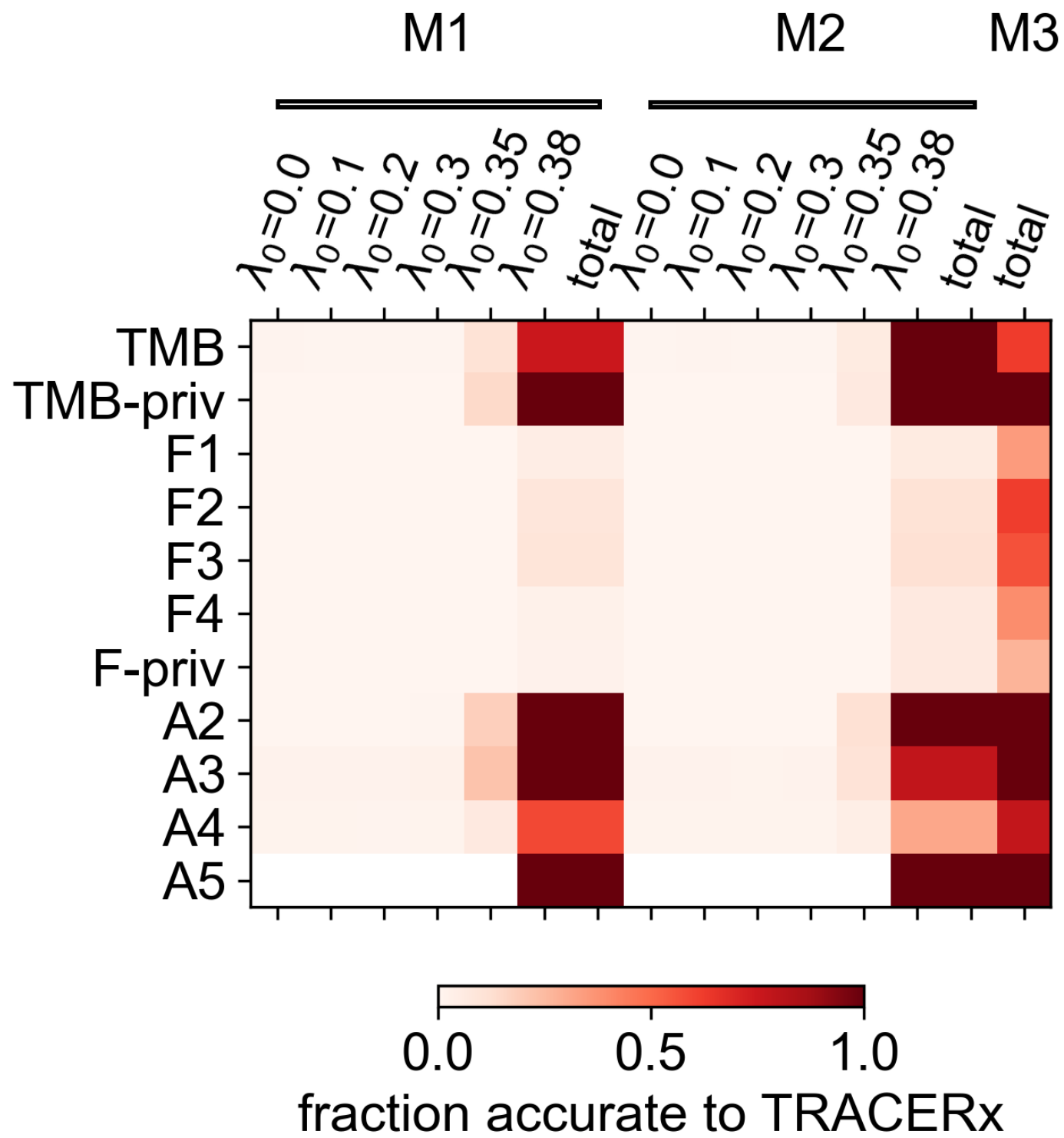

Supplementary Figure 17: A comparison of the suitability of all 3 models to the TRACERx data, assuming $M = 5$ samples.

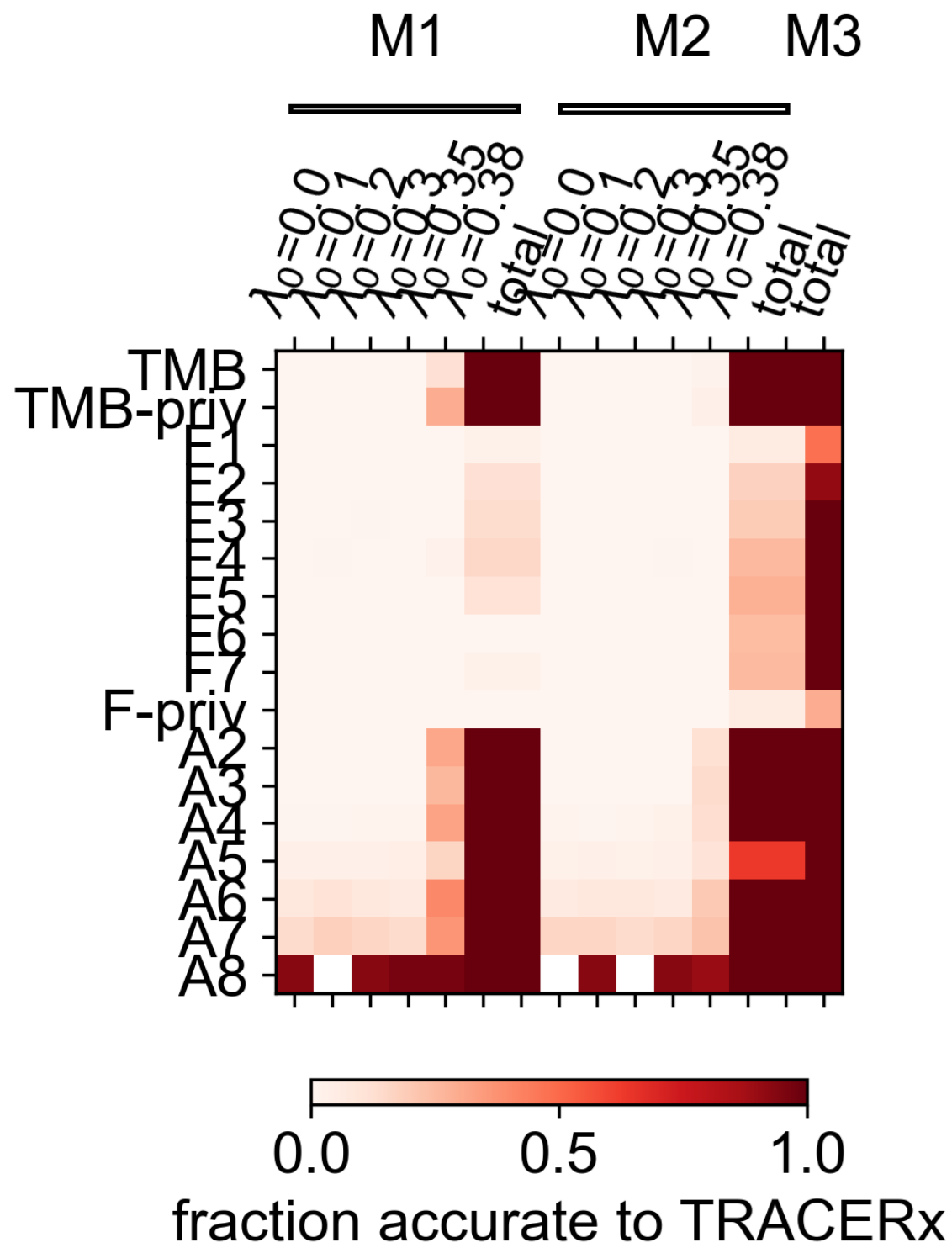

Supplementary Figure 18: A comparison of the suitability of all 3 models to the TRACERx data, assuming $M = 8$ samples.

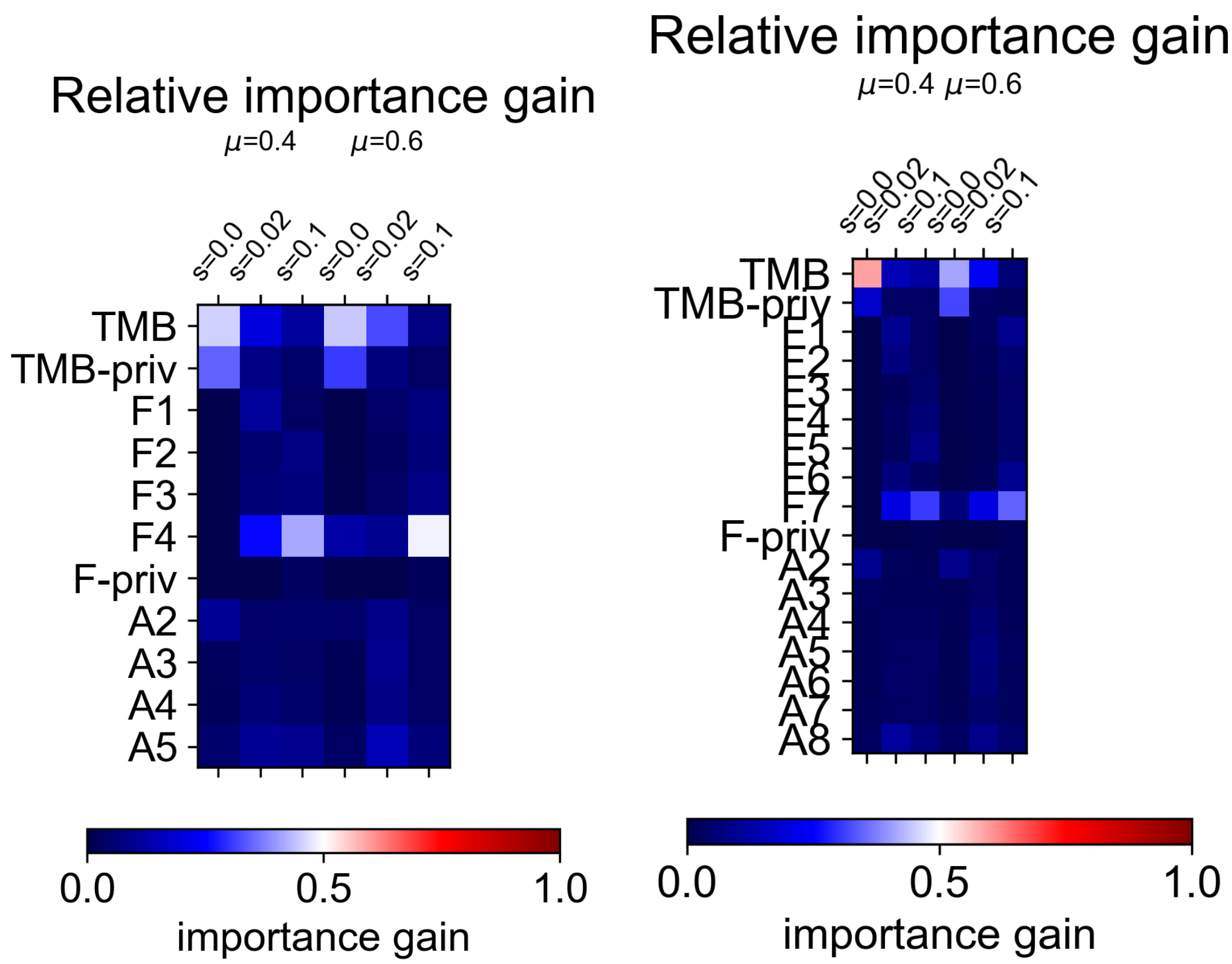

Supplementary Figure 19: The relative importance gain for each statistic, assuming $M = 5$ samples (left) or $M = 8$ samples (right). The (i, j)th element represents the relative importance gain of summary statistic i to the binary classifier responsible for recognising *in silico* tumors from scenario j. Importance gains are normalised across all summary statistics and 10 repeats of the classification process. The objective of XGB-Regressor is to minimize the MSE.

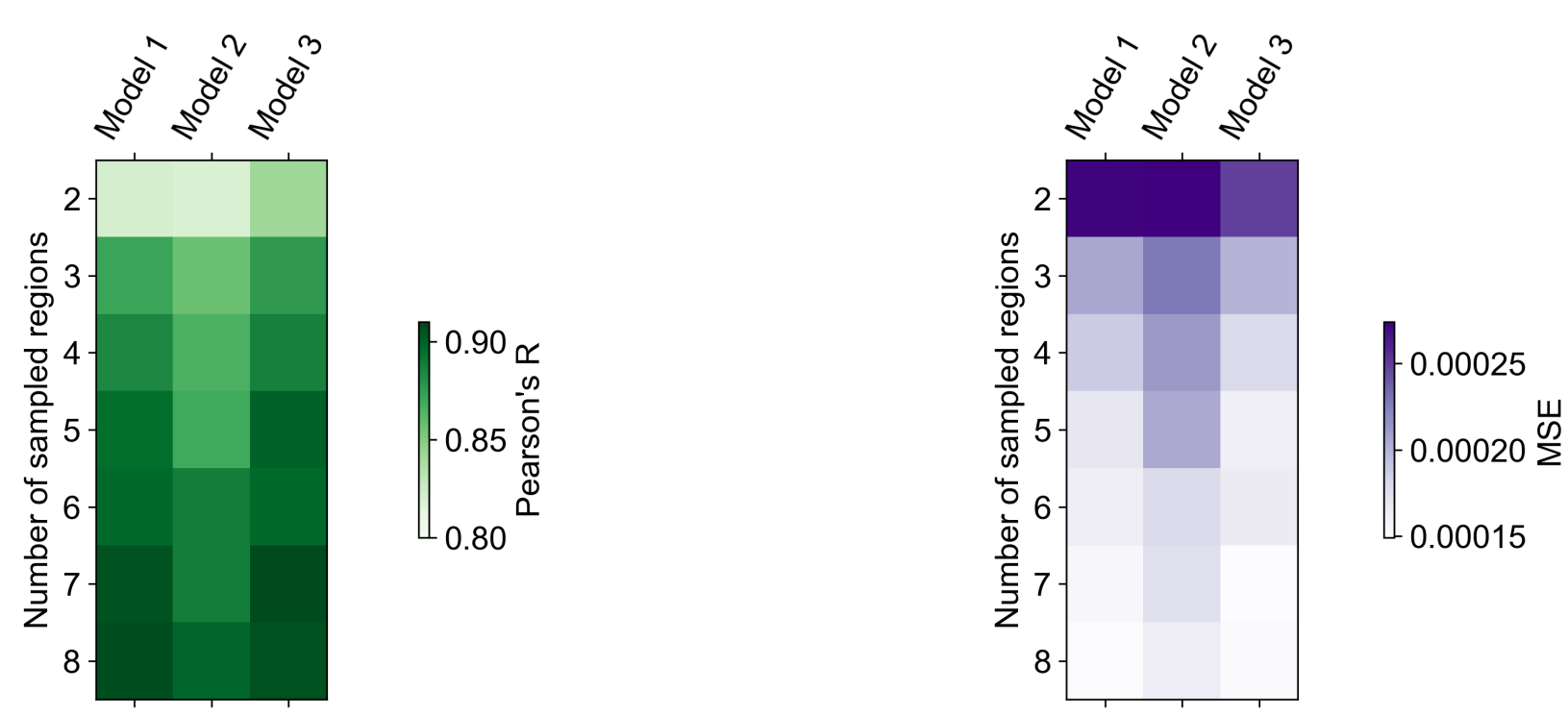

Supplementary Figure 20: The accuracy of inference from Models 1, 2, and 3, as measured by the correlation between true and inferred selection strength (Pearson's R, left) or mean squared error (right). Models 1 and 2 have an assumed death rate of $\lambda_0 = 0.38$. The inferred selection is taken as the average of the selection strengths corresponding to the 1% closest tested simulations; error bars indicate the standard deviations of these inferences.

	Clonal illusions (CIs)		Tumor mutational burden (TMB)	
	Mean (St. Dev.)	Median (IQR)	Mean (St. Dev.)	Median (IQR)
Tumor size (millions)				
2	14 (23)	6 (3-14)	117 (57)	94 (75-150)
5	19 (26)	10 (5-22)	138 (72)	116 (94-168)
10	28 (34)	14 (5-36)	160 (76)	138 (105-210)
20	44 (50)	24 (5-67)	189 (81)	180 (128-257)

Supplementary Table 1: The number of clonal illusions (CIs) and total observed non-truncal mutations (or total mutational burden, TMB) observed for Model 3 tumors run at high selection ($s = 0.1$), a mutation rate of $\mu = 0.4$ mutations per division, and varying tumor **size** (in millions of cells). Means, standard deviations, medians, and interquartile ranges are computed across 100 repeats.

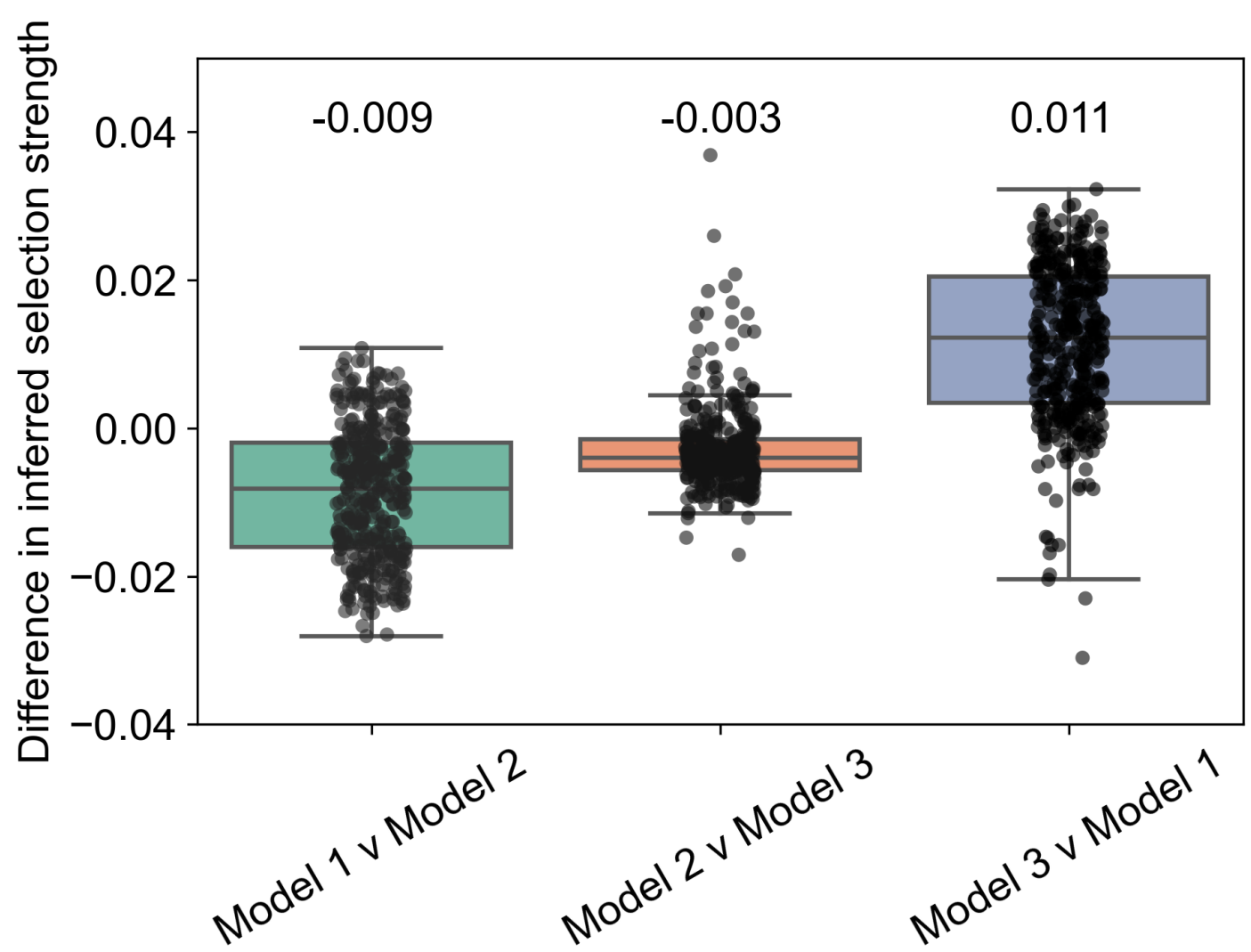

Supplementary Figure 21: A box plot of the pairwise differences between model inferences; each point represents the pairwise difference between mean model-specific estimates for the same tumor. The ith point in leftmost column corresponds to tumor i, and represents $s_{i1} - s_{i2}$, where s_{ij} is the average selection strength inferred from tumor i under Model j. The average of each distribution is given above each plot. Significance is tested using the distribution $s_{ij} - s_{ik}$, representing the difference in inferred selection levels for tumor i using Models j and k; we use a 1-sample T-test with the null-hypothesis that the mean of each distribution is zero, i.e. there is no significant difference between the predictions of Models j and k. All three p-values are of order 10^{-30} or lower.

References

[1] Sun R, Hu Z, Sottoriva A, Graham TA, Harpak A, Ma Z, et al. Between-region genetic divergence reflects the mode and tempo of tumor evolution. Nature Genetics. 2017 7;49:1015-24.

[2] Hu Z, Ding J, Ma Z, Sun R, Seoane JA, Shaffer JS, et al. Quantitative ev-

	Clonal illusions (CIs)		Tumor mutational burden (TMB)	
	Mean (St. Dev.)	Median (IQR)	Mean (St. Dev.)	Median (IQR)
Tumor age (years)				
0.5	11 (9)	8 (3-16)	107 (37)	107 (79-134)
1	16 (22)	8 (4-16)	136 (65)	117 (83-177)
1.5	21 (27)	11 (6-22)	138 (65)	125 (97-164)
2	26 (30)	15 (9-28)	150 (70)	127 (96-196)

Supplementary Table 2: The number of clonal illusions (CIs) and total observed non-truncal mutations (or total mutational burden, TMB) observed for Model 3 tumors run at high selection ($s = 0.1$), a mutation rate $\mu = 0.4$ mutations per division, and varying tumor **age**. Means, standard deviations, medians, and interquartile ranges are computed across 100 repeats.

idence for early metastatic seeding in colorectal cancer. Nature Genetics. 2019 7;51:1113-22.

[3] Hu Z, Li Z, Ma Z, Curtis C. Multi-cancer analysis of clonality and the timing of systemic spread in paired primary tumors and metastases. Nature Genetics. 2020 7;52:701-8.

[4] Zhao J, Siegmund KD, Shibata D, Marjoram P. Ancestral inference in tumors: How much can we know? Journal of Theoretical Biology. 2014 10;359:136-45.

[5] Williams MJ, Werner B, Heide T, Curtis C, Barnes CP, Sottoriva A, et al. Quantification of subclonal selection in cancer from bulk sequencing data. Nature Genetics. 2018 6;50:895-903.

[6] Zhao J, Salomon MP, Shibata D, Curtis C, Siegmund K, Marjoram P. Early mutation bursts in colorectal tumors. PLoS ONE. 2017 3;12.

[7] Frankell AM, Dietzen M, Bakir MA, Lim EL, Karasaki T, Ward S, et al. The evolution of lung cancer and impact of subclonal selection in TRACERx. Nature. 2023 4;616:525-33.

[8] Bakir MA, Huebner A, Martínez-Ruiz C, Grigoriadis K, Watkins TBK, Pich O, et al. The evolution of non-small cell lung cancer metastases in TRACERx. Nature. 2023 4;616:534-42.

[9] Toni T, Welch D, Strelkowa N, Ipsen A, Stumpf MPH. Approximate Bayesian computation scheme for parameter inference and model selection in dynamical systems. Journal of the Royal Society, Interface. 2009 2;6:187-202.

[10] Househam J, Heide T, Cresswell GD, Spiteri I, Kimberley C, Zapata L, et al. Phenotypic plasticity and genetic control in colorectal cancer evolution. Nature. 2022 11;611:744-53.

[11] Chkhaidze K, Heide T, Werner B, Williams MJ, Huang W, Caravagna G, et al. Spatially constrained tumour growth affects the patterns of clonal selection and neutral drift in cancer genomic data. PLoS Computational Biology. 2019 7;15.

[12] Siegmund KD, Marjoram P, Woo YJ, Tavaré S, Shibata D. Inferring clonal expansion and cancer stem cell dynamics from DNA methylation patterns in colorectal cancers. Proceedings of the National Academy of Sciences of the United States of America. 2009 3;106:4828-33.

[13] Jiang B, yu Wu T, Zheng C, Wong WH. Learning Summary Statistic for Approximate Bayesian Computation via Deep Neural Network. 2015 10. Available from: http://arxiv.org/abs/1510.02175 http://dx.doi.org/10.5705/ss.202015.0340.

[14] Grigoriadis K, Huebner A, Bunkum A, Colliver E, Frankell AM, Hill MS, et al. CONIPHER: a computational framework for scalable phylogenetic reconstruction with error correction. 2023. Available from: https://doi.org/10.21203/rs.3.pex-2158/v1.
